# Galacto-oligosaccharides alone and combined with lactoferrin impact the Kenyan infant gut microbiota and epithelial barrier integrity during iron supplementation *in vitro*

**DOI:** 10.20517/mrr.2024.34

**Published:** 2024-12-17

**Authors:** Carole Rachmühl, Christophe Lacroix, Adele Ferragamo, Ambra Giorgetti, Nicole U. Stoffel, Michael B. Zimmermann, Gary M. Brittenham, Annelies Geirnaert

**Affiliations:** ^1^Laboratory of Food Biotechnology, Institute of Food, Nutrition and Health, Department of Health Sciences and Technology, ETH Zürich, Zürich 8092, Switzerland.; ^2^Current address: Clean Mouse Facility, Experimental Animal Center, Department for Biomedical Research, University of Bern, Bern 3008, Switzerland.; ^3^Laboratory of Human Nutrition, Institute of Food, Nutrition and Health, Department of Health Sciences and Technology, ETH Zürich, Zürich 8092, Switzerland.; ^4^Medical Research Council Translational Immune Discovery Unit, Weatherall Institute of Molecular Medicine, John Radcliffe Hospital, University of Oxford, Oxford OX3 9DS, UK.; ^5^Department of Pediatrics, College of Physicians and Surgeons, Columbia University, New York, NY 10032, USA.

**Keywords:** Gut microbiome, *ex vivo* models, iron fortification, micronutrient, prebiotic, weaning infant

## Abstract

**Aim:** Iron supplementation to African weaning infants was associated with increased enteropathogen levels. While cohort studies demonstrated that specific prebiotics inhibit enteropathogens during iron supplementation, their mechanisms remain elusive. Here, we investigated the *in vitro* impact of galacto-oligosaccharides (GOS) and iron-sequestering bovine lactoferrin (bLF) alone and combined on the gut microbiota of Kenyan infants during low-dose iron supplementation.

**Methods:** Different doses of iron, GOS, and bLF were first screened during batch fermentations (*n* = 3), and the effect of these factors was studied on microbiota community structure and activity in the new Kenyan infant continuous intestinal PolyFermS model. The impact of different fermentation treatments on barrier integrity, enterotoxigenic *Escherichia coli* (ETEC) infection, and inflammatory response was assessed using a transwell co-culture of epithelial and immune cells.

**Results:** A dose-dependent increase in short-chain fatty acid (SCFA) production, *Bifidobacterium* and *Lactobacillus*/*Leuconostoc*/*Pediococcus* (LLP) growth was detected with GOS alone and combined with bLF during iron supplementation in batches. This was confirmed in the continuous PolyFermS model, which also showed a treatment-induced inhibition of opportunistic pathogens *C. difficile* and *C. perfringens*. In all tests, supplementation of iron alone and combined with bLF did not have a significant effect on microbiota composition and activity. We observed a strengthening of the epithelial barrier and a decrease in cell death and pro-inflammatory response during ETEC infection with microbiota fermentation supernatants from iron + GOS, iron + bLF, and iron + GOS + bLF treatments compared to iron alone.

**Conclusion:** Overall, beneficial effects on infant gut microbiota were shown using advanced *in vitro* models for GOS alone and combined with bLF during low-dose iron supplementation.

## INTRODUCTION

Iron deficiency anemia (IDA) is prevalent among African children under 5 years of age^[1]^, and high IDA prevalences of 62% to 73% were reported in Kenyan infants aged 6.5 to 12 months^[2,3]^. In-home fortification with complementary foods supplemented with iron-containing micronutrient powders (MNPs) is used to reduce IDA in African infants during weaning^[4,5]^. However, the safety of MNPs containing the WHO-recommended iron dose (10-12.5 mg/day for 6- to 23-month-old infants^[6]^) was questioned following reports about possible adverse effects on the infant gut microbiota^[7]^. Iron-induced gut microbial dysbiosis was previously reported for Kenyan and Pakistani infants and characterized by an increase of potential harmful bacteria (*Enterobacteriaceae*, pathogenic *E. coli*) and a decrease in beneficial taxa (*Bifidobacterium* and *Lactobacillus*)^[3,8-10]^.

Modified iron-containing MNPs with lower iron doses and compounds promoting beneficial gut microbes have been investigated to improve their safety and efficacy. The combination of iron with prebiotics galacto-oligosaccharides (GOS)^[3]^ or with a combination of GOS and fructo-oligosaccharides (FOS)^[11]^ resulted in improved iron absorption and positive effects on the gut microbiota composition in Kenyan infants during weaning. In both studies, the abundances of beneficial *Bifidobacterium* or *Lactobacillus* were higher, and the abundances of pathogens and toxin-encoding genes were lower in the feces of infants receiving prebiotics and iron compared to the iron group. The addition of the iron-binding protein bovine lactoferrin (bLF) has been suggested to further improve the safety of iron-containing MNPs. bLF is highly abundant in the whey protein fraction of milk^[12]^ and can promote iron absorption^[13]^. Other beneficial functions of lactoferrin include its bifidogenic^[14]^, antimicrobial^[15]^, and anti-inflammatory activities^[16]^. Formula fortified with bLF resulted in a significantly lower incidence of diarrhea in Chinese weaning infants^[17]^ and a lower prevalence of acute gastrointestinal symptoms in young Japanese children^[18]^. Additionally, bLF improved the iron absorption from a maize-based porridge containing FeSO_4_ and iron-free LF in Kenyan infants^[19]^. The efficacy of iron-containing MNPs with the prebiotic GOS and bLF to prevent IDA and microbial dysbiosis is investigated in Kenyan infants^[20]^.

Investigating MNP treatment effects on the gut microbiota in fecal samples does not enable a reliable assessment of the functional impact of the treatments, because intestinal-produced metabolites are largely absorbed and cannot be evaluated in feces^[21]^. *In vitro* gut microbiota models circumvent this limitation by investigating the gut microbiota independent of the host and under controlled conditions^[22]^. For example, the continuous fermentation model PolyFermS inoculated with immobilized fecal microbiota can test several treatments in parallel with the same complex human gut microbiota^[23,24]^, and can be combined with cellular models to study the host-microbe interactions^[25]^. The PolyFermS model was recently adapted to closely mimic the gut microbiota of Kenyan infants during weaning^[26]^. A strong prebiotic potential was confirmed for a short-chain GOS/long-chain FOS mixture and inulin but not for acacia gum in Kenyan infant gut microbiota during iron supplementation^[27]^.

The aim of this study was to investigate the direct effects of GOS and bLF, alone or combined, on both the microbe-microbe and host-microbe interactions in the gut microbiota of Kenyan infants during iron supplementation *in vitro*. First, the effect of different doses of iron as ferrous sulfate, GOS and bLF on the microbiota metabolic activity and growth of infant-characteristic bacteria was assessed during short-term batch fermentations inoculated with artificial microbiota produced from three different Kenyan infant PolyFermS models. Next, the effects of iron, GOS, bLF, and a combination thereof, each at concentrations selected to mimic a double-blind intervention study in Africa^[20]^ on microbiota community structure were investigated in continuous PolyFermS models inoculated with two different infant fecal microbiota. Finally, a transwell co-culture system of epithelial and immune cells was exposed to treated PolyFermS microbiota supernatants to investigate treatment effects on epithelial barrier integrity, pathogen infection and inflammatory response.

## METHODS

### Fecal donor characteristics and fecal sample collection

Fresh fecal samples from 5 infants aged between 5.6 and 9.7 months living in rural Kenya (Msambweni County) were collected, transported under protective anaerobic and cold conditions, and processed as previously described within less than 30 h for immobilization and inoculation of PolyFermS models^[26]^. None of the infants received antibiotics prior to sample donation and detailed donor information is given in Supplementary Table 1.

### *In vitro* gut microbiota fermentation experiments

**Figure 1:**
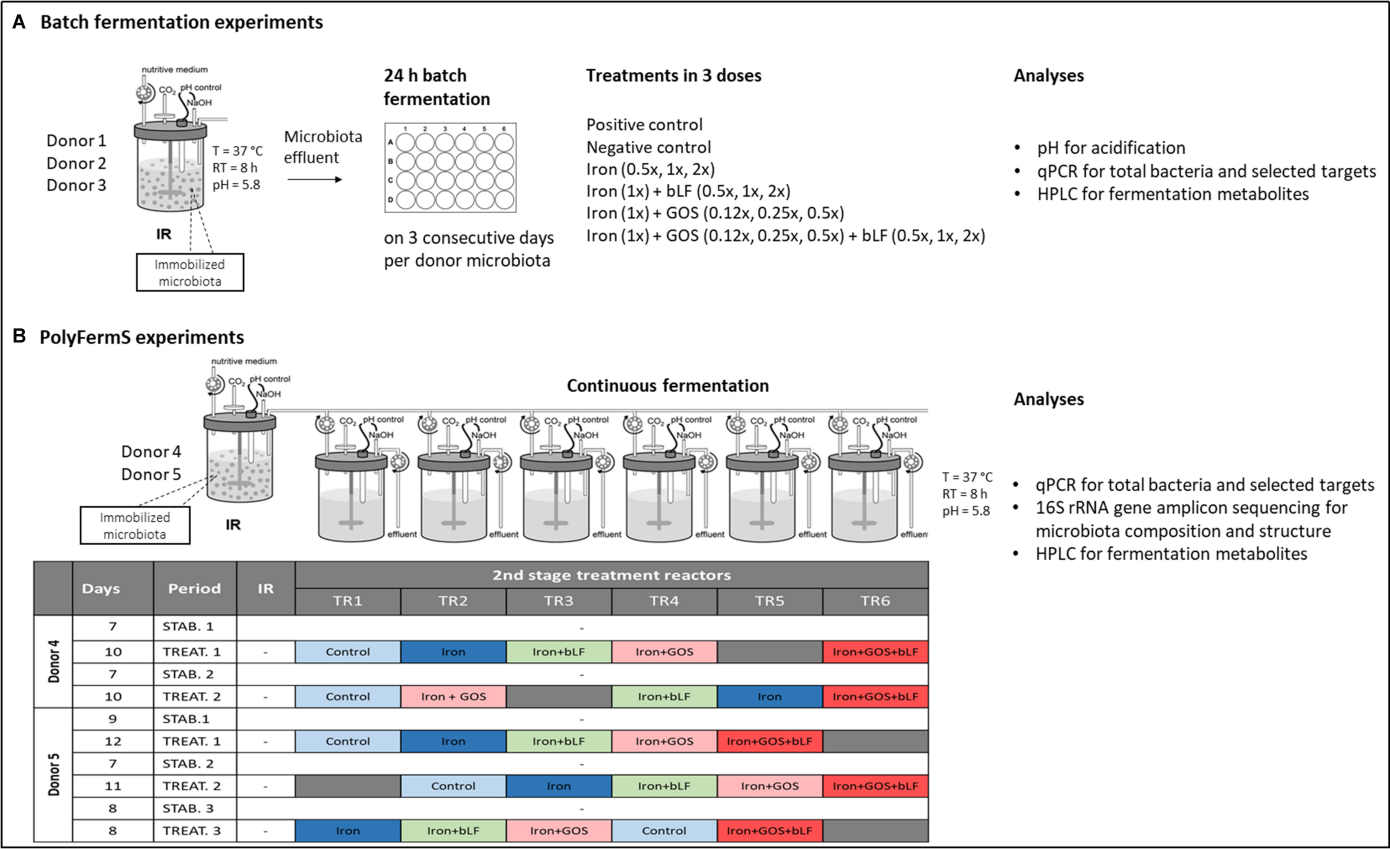
Overview of batch fermentation and PolyFermS experiments. IR with immobilized Kenyan infant fecal microbiota and connected PolyFermS second-stage treatment reactors (TR1-6). Each fecal microbiota was immobilized and cultivated separately. (A) IR microbiota of donors 1 to 3 were used for batch fermentations in 24-well plates, and (B) IR of donors 4 and 5 were connected to TRs. IR: Inoculum reactors; TRs: test reactors; qPCR: quantitative PCR; HPLC: high-performance liquid chromatography; T: temperature; RT: retention time; STAB: stabilization period; TREAT: treatment period.

The Kenyan infant PolyFermS model was used to continuously cultivate the donor fecal microbiota as previously described^[26]^. First, three PolyFermS Kenyan infant fecal microbiota (donor 1, 2 and 3) were used separately to assess the dose-dependent microbiota response to iron, GOS, and bLF during 24 h of batch fermentations in 24-well plates [Figure 1]. Next, the PolyFermS model was used to assess the effect of iron, GOS, and bLF on the microbiota community structure of two Kenyan infant fecal microbiota (donors 4 and 5) [Figure 1].

#### Cultivation medium

The cultivation medium was designed to mimic the ileal chyme entering the proximal colon of Kenyan infants during weaning at the age of 6 to 8 months and prepared as previously described^[26]^ and detailed in Supplementary Table 2. For batch fermentations, the medium composition was slightly adapted and contained a 6-fold higher concentration of sodium bicarbonate to enhance the internal buffering capacity. Additionally, carbohydrate sources were reduced by 50% to prevent over-acidification. Further, since phosphate has a strong capacity to bind iron and may affect iron solubility at high concentrations, potassium phosphate monobasic was substituted with 2-morpholinoethanesulfonic acid monohydrate (MES).

#### Fecal microbiota immobilization and PolyFermS model operation

Immobilization of fecal microbiota and PolyFermS bioreactor operation were performed as reported previously^[26]^ and detailed in Supplementary Materials. Each fecal microbiota was immobilized and cultivated in separate bioreactors, with details provided in Supplementary Materials. After bead colonization and stabilization for at least 21 days, the effluent from the bioreactor with immobilized fecal microbiota served as a microbial inoculum source for batch fermentations. The inoculum reactors (IR) of donors 4 and 5 were connected to second-stage test reactors (TRs) after stabilization, as reported previously^[27]^. TRs were continuously inoculated with IR effluent (1.25 mL/h) and simultaneously fed with fresh cultivation medium (23.75 mL/h). The operation conditions of IR and TRs were the same, except for stirring, which was 120 rpm for IR and 180 rpm for TR [Figure 1]. TRs were operated for at least 7 days after connection and prior to treatment start, to reach a pseudo-steady state monitored through a day-to-day variation in short-chain fatty acid (SCFA) production of less than 10%^[28]^.

Effluent samples of IR and TRs were collected daily for metabolite analysis with high-performance liquid chromatography (HPLC). The samples (2 mL) were centrifuged for 10 min at 18,407 *g* and 4 °C. Supernatants were used for HPLC analyses and pellets stored at -80 °C for DNA extraction.

#### Batch fermentation and PolyFermS experimental setup

Iron (FeSO_4_·7H_2_O; Sigma-Aldrich), GOS (Vivinal, 70% GOS, 24% lactose, 6% glucose + galactose; Friesland Campina), and iron-free apo-bLF (Vitalarmor Lactoferrin, Armor Protéines) were used. The treatments were set to mimic an infant's daily oral dose of 5 mg elemental iron, 10 g of Vivinal GOS, and 1 g of apo-bLF, respectively^[20]^. Therefore, 24.9 mg FeSO_4_·7H_2_O/L medium (5 mg elemental iron/L), 11.1 g Vivinal GOS/L medium, and 1.1 g apo-bLF/L medium were used for the 1× treatments in 24-well plates and for the PolyFermS experiments. Dosage calculations considered an estimated infant proximal colon capacity of 300 mL^[29]^, 8 h retention time^[30]^, and 10% iron absorption in the small intestine, leading to 90% of iron entering the proximal colon^[26,31,32]^.

Batch fermentations were performed over three consecutive days using fresh effluent microbiota derived from the IR of donors 1, 2, and 3 to screen different treatment doses [Figure 1A]. Iron and bLF were added to the medium at concentrations simulating the *in vivo* (1×) dose as described above, and half (0.5×) and double (2×) concentrations. In contrast, the Vivinal GOS dose effect was tested at lower levels of 0.12×, 0.25×, and 0.5× to maintain pH above 5.0, because higher concentrations led to strong acidification. Effluent samples were collected in N_2_-flushed serum flasks and transferred immediately to an anaerobic chamber (Coy Laboratory Products, USA; 10% CO_2_, 5% H_2_ and 85% N_2_). Diluted microbiota (10% v/v in anaerobic peptone water, pH 7.0) were then inoculated at 1% (v/v) in wells containing 2 mL medium with the supplemented treatments, in technical duplicate (donor 1 and 2 IR microbiota) or triplicate (donor 3 IR microbiota) per consecutive day repeat. Inoculated wells containing non-supplemented cultivation medium served as controls for the treatments, while non-inoculated wells served as negative controls. The plates were incubated for 24 h at 37 °C (INCU-Line 10 digital incubator, VWR International AG) in the anaerobic chamber. Subsequently, 1 mL of each well was centrifuged for 10 min at 18,407 *g* and 4 °C. The supernatant was used for HPLC analysis, and the sample pellet was stored at -80 °C for DNA extraction. The pH was measured (pH meter, Metrohm Switzerland Ltd) using the remaining 1 mL of the sample.

The effect of 1× dose treatments was assessed using PolyFermS model TRs of donors 4 and 5 in two and three repetitions, respectively [Figure 1B]. FeSO_4_·7H_2_O was added to the medium components prior to dissolution in dH_2_O. Vivinal GOS and bLF were dissolved in dH_2_O separately, filter-sterilized (0.2 µm) and supplemented to the cultivation medium after autoclaving. The iron concentration was measured with Inductively Coupled Plasma - Mass Spectrometry iCap, KED mode in the non-supplemented (2.26 ± 0.05 mg/L) and supplemented (7.64 ± 0.38 mg/L) medium as previously described^[33]^. Between experimental periods, TRs were disconnected, cleaned, autoclaved, reconnected, and re-stabilized for 7 to 8 days before testing a new treatment. Treatments were randomized among TRs during each period to prevent possible reactor effects. Effluent samples of the last three days of stabilization and the last three days of treatment were processed as described above for the different analyses.

### Molecular analysis

The FastDNA SPIN Kit for Soil (MP Biomedicals) was used to extract the DNA of fecal (200 mg) and effluent (pellet of 2 mL) samples according to the manufacturer’s instructions.

Quantitative PCR (qPCR) was performed to determine the absolute numbers of total and selected bacterial targets of the infant gut microbiota (primer details in Supplementary Table 3). Reactions were performed using the Roche Light Cycler 480 System (Hoffmann-La Roche) as previously described^[26]^. The *qPCR* gene copy number was adjusted for the median number of 16S rRNA gene copies of each target using the Ribosomal RNA Database^[34]^ to convert the data into absolute bacterial concentrations.

The Illumina MiSeq platform was used to perform paired-end 16S rRNA gene amplicon sequencing (Illumina) at the Genetic Diversity Center (GDC, ETH Zurich) as previously described^[26]^. The V4 region of the 16S rRNA gene was amplified with the primer combination nxt_515F/nxt_806R (5’-GTGCCAGCMGCCGCGGTAA-3’, 5’-GGACTACHVGGGTWTCTAAT-3’) followed by amplicon barcoding using Nextera Index primers. The DADA2-pipeline^[35,36]^ was used to generate amplicon sequencing variants (ASV) as previously described^[26]^. Forward and reverse reads were truncated after 170 nucleotides and 160 nucleotides, respectively. Truncated reads with an expected error rate higher than three for forward and four for reverse reads were removed. After filtering, denoising, error rate learning, and ASV inference, reads were merged with a minimum overlap of 40 bp. Chimeric sequences were removed, and taxonomy was assigned using the SILVA database (v.132)^[37]^.

### Metabolite analysis

SCFA (acetate, propionate, butyrate, and valerate), branched-chain fatty acids (BCFA, isobutyrate, isovalerate), and intermediate metabolites (succinate, lactate, and formate) were quantified in fecal (200 mg) and effluent (2 mL) samples using HPLC as previously described^[26]^.

### Mammalian cell model for microbe-host experiments

To mimic the gut mucosal environment, a transwell plate system was used to co-culture intestinal epithelial Caco-2 (DSMZ ACC 169) and mucin-producing HT29-MTX (ECACC 12040401) cells in the apical and THP-1 Blue cells (Invivogen, thp-nfkb) in the basolateral compartment. THP-1 Blue cells were transfected with a nuclear factor kappa B (NFκB)-inducible secreted embryonic alkaline phosphatase (SEAP) reporter construct (Invivogen), which enables the screening of pro-inflammatory NFκB activation. For simulating pathogen infection, enterotoxigenic *Escherichia coli* (ETEC) was chosen due to its high prevalence in Kenyan infants (21%-49.2%) and other countries of sub-Saharan Africa^[3,9,38]^.

#### Bacterial strain and culture conditions

ETEC strain H10407 (ATCC 35401, LGC Standards GmbH) was routinely grown in fresh Luria-Bertani (LB) broth Miller (Becton Dickinson AG) at 37 °C and shaken at 120 rpm (Adolf Kühner AG). ETEC was grown to an optical density of 0.5 (approximately 1.4 × 10^8^ CFU/mL), centrifuged (10 min at 18,407 *g*), and washed once in phosphate-buffered saline (PBS, Thermo Fisher Scientific) prior to resuspension in minimum essential medium (MEM) Hanks’ Balanced Salts (Thermo Fisher Scientific) for infection experiments.

#### Co-culture model of epithelial and immune cells

Caco-2 and HT29-MTX cells were cultivated as described in Supplementary Materials. Cells were seeded on cell insert membranes (0.4 µm) of a Millicell 24-well cell culture insert plate (Sigma-Aldrich) in a 75:25 ratio^[25]^ at a final concentration of 5.0 × 10^4^ cells/cm^2^ and cultivated for 16 to 21 days to reach full differentiation. The medium was exchanged every 2 days in the apical and basolateral compartments of the transwell plate. THP-1 Blue cells were seeded (in RPMI 1640 HEPES medium without antibiotics) into the basolateral compartment at a final concentration of 8.0 × 10^4^ cells/well 24 h prior to combination with the apical insert plate containing the fully differentiated Caco-2/HT29-MTX cell monolayer.

#### Treatment with PolyFermS microbiota supernatants and infection experiments

**Figure 2:**
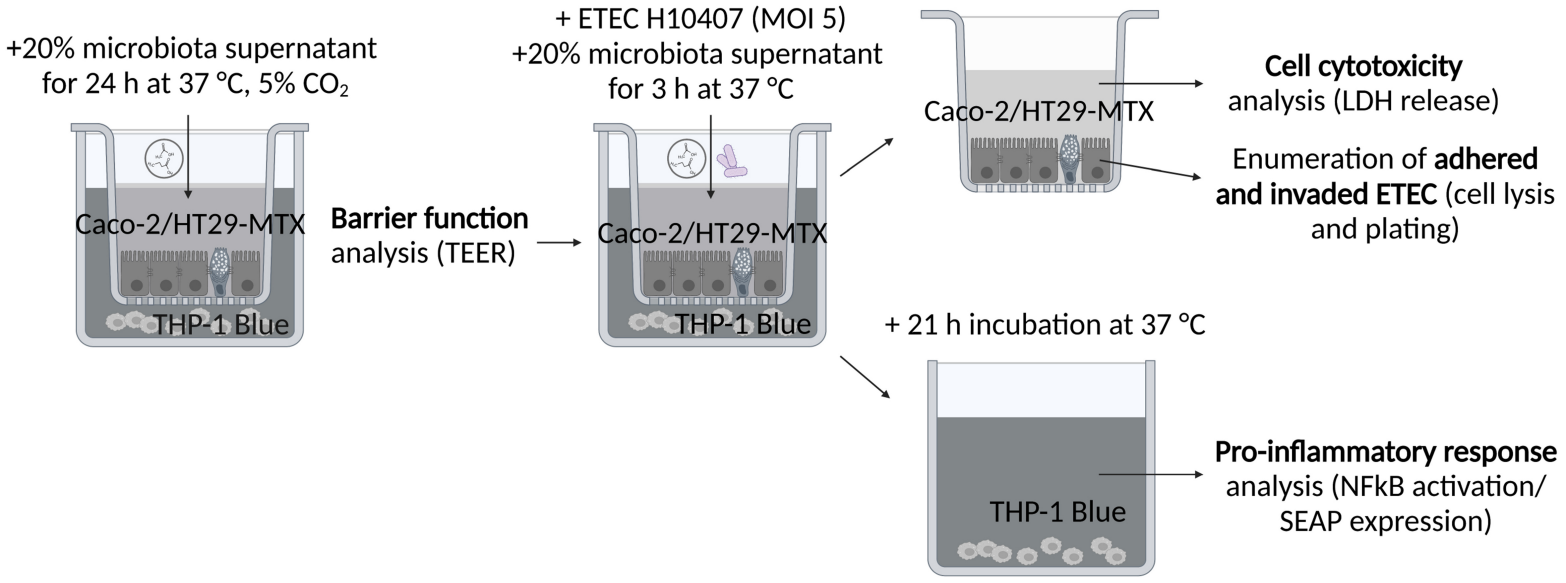
Overview of mammalian cell model setup using a transwell system to assess the impact of differently treated PolyFermS supernatant on the epithelial barrier, pathogen infection, and inflammatory response. TEER: Transepithelial electrical resistance; MOI: multiplicity of infection; LDH: lactate dehydrogenase; ETEC: enterotoxigenic *Escherichia coli*; NFκB: nuclear factor kappa B; SEAP: secreted embryonic alkaline phosphatase.

Caco-2/HT29-MTX cell monolayers were exposed to PolyFermS supernatant from control and treated donor 4 and donor 5 microbiota (last treatment day of period 1 and 2, respectively) to assess its impact on barrier integrity and infected cell model with ETEC [Figure 2].

Effluent samples were centrifuged (15 min at 18,407 *g*, 4 °C) and the supernatant was filter-sterilized (0.2 µm). To assess the residual iron concentration in the supernatants, the total elemental iron was determined with the ferrozine assay and detailed in Supplementary Figure 1. To prevent osmotic stress to the cells, supernatant osmolality was adjusted to 300 mOsm/kg with dH_2_O (Vapro Vapor Pressure Osmometer, ELITechGroup) before addition to antibiotic-free Dulbecco’s modified Eagle’s Medium (DMEM, 20%, v/v). After 24 h incubation of cells with supernatant-containing DMEM (37 °C, 5% CO_2_), barrier integrity was assessed by measuring transepithelial electrical resistance (TEER) in triplicate per well using a Millicell-ERS Voltohmmeter (Merck&Cie). Subsequently, the apical medium was replaced by MEM (Hank’s Balanced Salts, made for use without CO_2_) containing supernatant (20%, v/v), and ETEC was added at a final concentration of 1.4 × 10^6^ bacteria/well (approximate multiplicity of infection of 5). After 3 h of incubation at 37 °C (non-humified incubator), the supernatant of Caco-2/HT29-MTX cells was used to measure the release of lactate dehydrogenase (LDH), a marker for cell cytotoxicity. The supernatant was collected and centrifuged for 10 min at 18,407 *g*. LDH concentration in the bacteria-free supernatant was measured using the CytoTox 96 Non-Radioactive Cytotoxicity Assay according to the manufacturer’s instructions (Promega). To enumerate adhered and invaded ETEC, Caco-2/HT29-MTX cells were washed twice with PBS and disrupted using 0.1% Triton X-100 (VWR International). Serial tenfold dilutions of disrupted cells were plated on LB Miller agar. Agar plates were incubated overnight at 37 °C for the enumeration of viable ETEC. The basolateral compartment containing THP-1 Blue cells was incubated for another 21 h at 37 °C prior to assessment of the pro-inflammatory response. SEAP expression in the supernatant of THP-1 Blue cells was assessed spectrophotometrically at OD 620 nm using QUANTI-BLUE according to the manufacturer’s instructions (Invivogen, Labforce). The experiments were performed using three independent cell passages with technical duplicates. TEER before the start of experiments ranged from 396 to 560 Ω·cm^2^.

### Statistical analysis and data visualization

Microbiota community analysis was done in R (version 4.0.4) using the phyloseq^[39]^, vegan^[40]^, and ggplot2^[41]^ packages. Differential relative abundance analysis was performed using DESeq2^[42]^. Rarefied data were used to calculate relative abundances, as well as alpha and beta diversity. GraphPad Prism (v 9.1.0) was used to create graphs and for statistical analysis. Normal distribution was assessed using the Shapiro-Wilk test. A paired *t*-test was applied to test differences between two independent normal-distributed samples. Welch’s test was used in case of unequal variances and the Mann-Whitney test was applied for samples that were not normally distributed. Differences between more than two independent normally distributed samples were tested with one-way ANOVA, and in case of statistical significance, a Dunnet’s post-hoc test was performed. The Kruskal-Wallis test was used for samples that were not normally distributed with a post hoc Dunn’s test in case of statistical significance. The statistical level of significance was set to *P* < 0.05.

## RESULTS

### Dose-dependent microbiota response to iron, GOS, and bLF supplementation during batch fermentation

The response to different doses of iron, GOS, and bLF was assessed during 24 h batch fermentations in 24-well plates inoculated with three different Kenyan infant PolyFermS microbiota [Supplementary Figure 2], and key infant gut bacterial taxa growth and metabolite production were evaluated.

Iron supplementation with GOS, alone or combined with bLF, stimulated *Bifidobacterium* and *Lactobacillus*/*Leuconostoc*/*Pediococcus* (LLP) [Table 1], increased total metabolic activity [Table 2] and consequently decreased the final pH by about 1.5 pH unit at the highest GOS dose [Supplementary Table 4] compared to iron (1×) alone in all three microbiota. GOS response increased with the dose (*P* < 0.05) for LLP and for total metabolic activity. For microbiota 1, the production of acetate, propionate, and butyrate increased with the GOS level, while for microbiota 2, mainly acetate and formate, and for microbiota 3, acetate, formate, succinate, and lactate responded to GOS [Table 2 and Supplementary Table 6]. Growth of potential pathogenic taxa *Clostridioides difficile*, *Clostridium perfringens*, and Enteropathogenic *Escherichia coli* were not affected by the treatments in all three microbiota [Supplementary Table 5].

**Table 1:** Quantification of key bacterial taxa in Kenyan infant PolyFermS microbiota treated with iron, GOS and bLF during 24 h batch fermentations in 24-well plates

		***Bifidobacterium* (bacteria/mL)**			**LLP (bacteria/mL)**		
		**Donor 1**	**Donor 2**	**Donor 3**	**Donor 1**	**Donor 2**	**Donor 3**
**log_10_**	Inoculum	9.70 ± 0.31	8.99 ± 0.08	ND	9.10 ± 0.08	8.73 ± 0.36	ND
	Control	7.63 ± 0.23	9.30 ± 0.27	8.24 ± 0.11	7.43 ± 0.06	7.89 ± 0.26	6.24 ± 0.56
**Δlog_10_ compared to control**	Iron 0.5×	-0.12 ± 0.50	0.02 ± 0.03	-0.09 ± 0.09	0.09 ± 0.05	0.15 ± 0.30	0.16 ± 0.18
	Iron 1×	0.06 ± 0.57	0.05 ± 0.09	0.00 ± 0.08	0.15 ± 0.12	0.02 ± 0.07	0.29 ± 0.12
	Iron 2×	-0.17 ± 0.42	-0.19 ± 0.12	-0.03 ± 0.08	0.00 ± 0.11	-0.14 ± 0.12	0.19 ± 0.17
	Iron 1× bLF 0.5×	-0.06 ± 0.28	0.11 ± 0.15	-0.07 ± 0.07	0.01 ± 0.11	0.01 ± 0.00	0.17 ± 0.12
	Iron 1× bLF 1×	0.05 ± 0.48	0.06 ± 0.06	-0.10 ± 0.13	0.19 ± 0.05	0.08 ± 0.05	0.22 ± 0.27
	Iron 1× bLF 2×	-0.11 ± 0.55	-0.09 ± 0.07	-0.09 ± 0.13	0.08 ± 0.19	0.01 ± 0.09	0.11 ± 0.28
	Iron 1× GOS 0.12×	-0.01 ± 0.10	0.16 ± 0.07	0.23 ± 0.11	0.08 ± 0.06	-0.07 ± 0.24	0.38 ± 0.10
	Iron 1× GOS 0.25×	0.03 ± 0.46	0.43 ± 0.09^***^	0.39 ± 0.17	0.26 ± 0.19	0.20 ± 0.04	0.71 ± 0.23
	Iron 1× GOS 0.5×	0.43 ± 0.66	0.47 ± 0.06^***^	0.59 ± 0.24^*^	0.51 ± 0.09^*^	0.44 ± 0.15^*^	1.11 ± 0.21^**^
	Iron 1× bLF 0.5× GOS 0.12×	0.07 ± 0.09	0.22 ± 0.09	0.17 ± 0.11	0.01 ± 0.11	0.19 ± 0.10	0.33 ± 0.14
	Iron 1× bLF 1× GOS 0.25×	0.13 ± 0.18	0.43 ± 0.12^**^	0.35 ± 0.19^*^	0.20 ± 0.15	0.33 ± 0.04^**^	0.55 ± 0.04^*^
	Iron 1× bLF 2× GOS 0.5×	0.33 ± 0.23^*^	0.46 ± 0.22^**^	0.49 ± 0.22^*^	0.29 ± 0.06^*^	0.52 ± 0.14^***^	0.74 ± 0.15^**^

Mean ± SD of log_10_ bacteria/mL effluent is shown for inoculum (IR effluent) and control. The difference between treatments and control was reported as mean ± SD of Δlog_10_ bacteria/mL effluent. *n* = 3 repeats per donor with inoculum derived from 3 consecutive fermentation days. Significant differences between treatments with GOS or bLF compared to iron 1× are indicated by ^*^P < 0.05, ^**^P < 0.01, ^***^P < 0.001, ^****^P < 0.0001. GOS: Galacto-oligosaccharides; bLF: bovine lactoferrin; LLP: *Lactobacillus*/*Leuconostoc*/*Pediococcus*; ND: not determined; IR: inoculum reactors.

**Table 2:** Quantification of total metabolites and SCFA in Kenyan infant PolyFermS microbiota treated with iron, GOS and bLF during 24 h batch fermentations in 24-well plates

		**Total metabolites (mM)**			**Acetate (mM)**			**Propionate (mM)**			**Butyrate (mM)**			**Formate (mM)**		
		**Donor 1**	**Donor 2**	**Donor 3**	**Donor 1**	**Donor 2**	**Donor 3**	**Donor 1**	**Donor 2**	**Donor 3**	**Donor 1**	**Donor 2**	**Donor 3**	**Donor 1**	**Donor 2**	**Donor 3**
**mM**	Inoculum	108.39 ± 4.28	96.10 ± 4.06	ND	57.68 ± 0.60	52.62 ± 1.80	ND	27.79 ± 4.17	27.25 ± 2.55	ND	5.39 ± 0.65	9.81 ± 0.49	ND	16.14 ± 13.96	4.25 ± 0.09	ND
	Control	88.24 ± 9.18	81.30 ± 6.57	86.90 ± 1.33	51.88 ± 13.93	38.35 ± 4.97	49.48 ± 3.32	8.98 ± 1.52	19.97 ± 1.26	4.10 ± 0.56	12.20 ± 1.51	5.75 ± 1.94	6.76 ± 0.39	20.91 ± 5.64	13.66 ± 1.90	18.75 ± 3.94
**ΔmM compared to control**	Iron 0.5×	-4.70 ± 5.11	-0.36 ± 3.87	-1.24 ± 3.07	-4.04 ± 2.60	-1.15 ± 1.83	-1.78 ± 0.56	0.38 ± 0.40	-0.49 ± 0.46	-0.18 ± 0.13	-0.71 ± 0.37	0.20 ± 0.51	-0.94 ± 0.25	-0.15 ± 1.19	0.87 ± 0.33	1.03 ± 2.35
	Iron 1×	-4.59 ± 1.98	-1.03 ± 2.41	-2.09 ± 2.93	-5.70 ± 4.37	-1.68 ± 1.56	-0.76 ± 0.49	0.63 ± 1.32	-0.17 ± 0.31	-0.15 ± 0.23	-0.52 ± 0.43	0.38 ± 0.66	-1.06 ± 0.39	-0.97 ± 1.60	0.68 ± 0.94	-0.50 ± 2.09
	Iron 2×	-2.29 ± 4.23	0.11 ± 1.68	-0.20 ± 2.13	-2.18 ± 2.70	-0.56 ± 0.35	1.17 ± 0.98	1.26 ± 1.12	0.46 ± 0.43	-0.03 ± 0.24	-0.44 ± 0.93	0.69 ± 0.84	-0.61 ± 0.13	-0.90 ± 1.61	-0.17 ± 0.46	-1.15 ± 1.83
	Iron 1× bLF 0.5×	-6.70 ± 4.62	-3.26 ± 2.42	-3.61 ± 2.26	-5.35 ± 7.98	-1.72 ± 1.50	-1.27 ± 0.36	0.64 ± 0.75	-0.52 ± 0.33	0.17 ± 0.14	-1.00 ± 0.08	-0.49 ± 1.11	-1.48 ± 0.28	-0.71 ± 4.59	-0.24 ± 1.25	-1.73 ± 1.82
	Iron 1× bLF 1×	-7.34 ± 4.69	-1.72 ± 2.29	-2.57 ± 2.10	-5.36 ± 9.19	-1.59 ± 1.19	-1.02 ± 0.46	0.99 ± 1.50	-0.24 ± 0.05	0.27 ± 0.10^*^	-1.42 ± 0.14^*^	-0.19 ± 0.88	-1.87 ± 0.31^*^	-1.24 ± 6.13	0.44 ± 1.35	-1.02 ± 1.73
	Iron 1× bLF 2×	-1.39 ± 4.33	3.74 ± 3.96	-0.16 ± 2.68	-0.27 ± 6.29	1.44 ± 2.79	2.93 ± 0.20^***^	1.74 ± 1.13	0.96 ± 0.56^*^	1.00 ± 0.14^****^	-1.58 ± 0.69	0.53 ± 1.15	-2.42 ± 0.34^**^	-1.36 ± 3.83	1.01 ± 1.18	-3.47 ± 2.05
	Iron 1× GOS 0.12×	8.24 ± 2.53^**^	13.46 ± 2.02	8.01 ± 2.38	2.30 ± 1.42	8.10 ± 1.71 *	-1.58 ± 1.02	1.27 ± 0.20	1.64 ± 0.35	-0.31 ± 0.66	2.96 ± 0.63	1.03 ± 0.38	-0.39 ± 0.75	1.16 ± 1.57	2.43 ± 0.58	8.68 ± 2.57
	Iron 1× GOS 0.25×	16.67 ± 4.45^***^	23.95 ± 3.92	19.52 ± 4.33^**^	2.13 ± 2.51	18.41 ± 4.30^***^	-0.29 ± 0.46	3.74 ± 0.80	3.01 ± 1.17	-1.01 ± 0.43	6.05 ± 1.02^**^	0.82 ± 0.88	0.18 ± 0.77	4.10 ± 3.08	1.85 ± 2.03	16.21 ± 4.06
	Iron 1× GOS 0.5×	30.11 ± 4.28^****^	45.95 ± 6.20^**^	37.59 ± 7.64^***^	5.81 ± 5.90^*^	30.42 ± 6.31^****^	3.80 ± 0.14^**^	5.48 ± 2.44^**^	9.80 ± 2.81^*^	-2.37 ± 0.61^***^	10.80 ± 3.25^***^	0.36 ± 3.62	0.25 ± 0.74	7.46 ± 5.76	5.83 ± 4.39	24.89 ± 6.26^**^
	Iron 1× bLF 0.5× GOS 0.12×	2.75 ± 0.64^*^	7.06 ± 1.32	7.50 ± 1.38	-3.30 ± 3.32	4.26 ± 1.94	-1.79 ± 1.51	1.45 ± 0.86	1.19 ± 0.84	-0.04 ± 0.55	2.46 ± 1.07	0.15 ± 0.72	-0.71 ± 0.47	2.56 ± 1.81	1.52 ± 1.31	8.07 ± 0.67^*^
	Iron 1× bLF 1× GOS 0.25×	13.36 ± 2.52^****^	19.91 ± 1.04	**19.28 ± 3.10**	0.57 ± 2.69	14.89 ± 5.27^**^	-0.06 ± 1.93	2.90 ± 0.12	3.48 ± 0.58	-0.83 ± 0.41	5.53 ± 1.50	0.53 ± 1.78	-0.39 ± 0.67	4.56 ± 3.32	0.99 ± 2.48	15.72 ± 2.86^***^
	Iron 1× bLF 2× GOS 0.5×	33.93 ± 2.81^****^	42.90 ± 1.18^**^	37.18 ± 7.46^***^	9.59 ± 3.69^**^	30.90 ± 6.53^****^	7.09 ± 1.77^**^	3.96 ± 3.25^*^	9.72 ± 2.26^*^	-2.11 ± 0.53^***^	10.54 ± 2.32^**^	0.15 ± 3.76	0.36 ± 0.33	9.34 ± 10.05	2.61 ± 4.68	22.50 ± 5.19^****^

Mean ± SD of metabolite concentration (mM) is shown for inoculum (effluent) and control. The difference between treatments and control was calculated and is shown as mean ± SD of Δ metabolite concentration (ΔmM). *n* = 3 repeats per donor with inoculum derived from 3 consecutive fermentation days. Significant differences between treatments with GOS or bLF and iron 1× are indicated by ^*^P < 0.05, ^**^P < 0.01, ^***^P < 0.001, ^****^P < 0.0001. SCFA: Short-chain fatty acid; GOS: galacto-oligosaccharides; bLF: bovine lactoferrin; ND: not determined.

### GOS supplementation stimulates SCFA production and growth of beneficial gut microbes while inhibiting potential pathogens during long-term continuous cultivation

Two independent continuous PolyFermS models inoculated with gut microbiota from Kenyan infants 4 and 5 were used to directly test the effect of 1× iron, GOS, and bLF supplementation on the microbiota community structure, dynamics, and metabolite production.

The composition and metabolite profile of the two PolyFermS microbiota after initial colonization and stabilization differed [Supplementary Figures 3 and 4]. PolyFermS microbiota of donor 4 was dominated by *Bifidobacterium*, *Streptococcus*, *Lactococcus*, and *Veillonella*, which was akin to the fecal microbiota, and produced high concentrations of acetate, propionate, and formate. PolyFermS microbiota of donor 5 was dominated by *Bacteroides*, *Streptococcus*, and *Megasphaera* and produced high concentrations of acetate and butyrate. All parallel reactors of donor 4 or donor 5 PolyFermS microbiota exhibited a similar baseline at the end of the stabilization periods of 7 to 9 days, enabling the evaluation of different treatment combinations on a similar microbiota.

**Figure 3:**
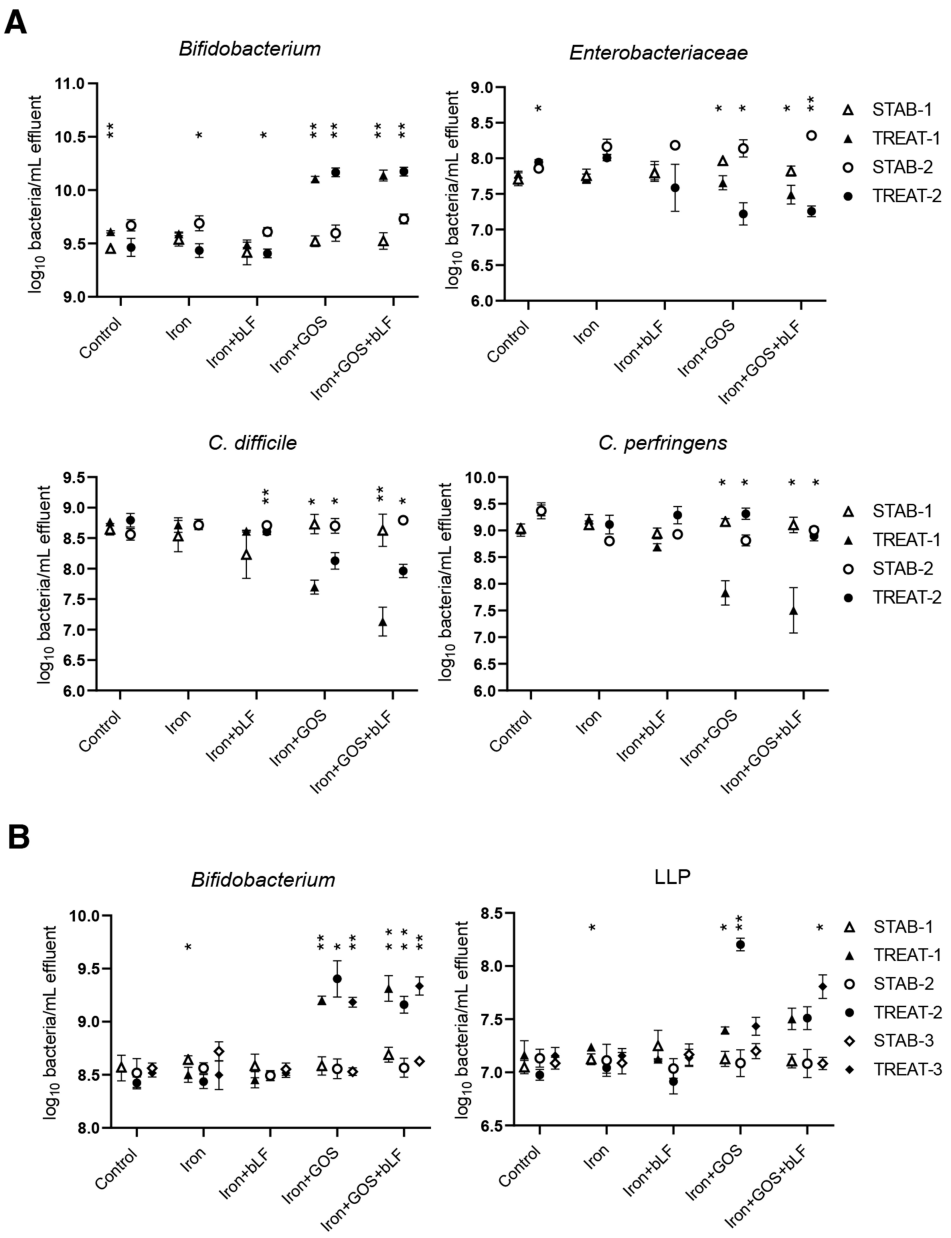
Quantification of beneficial and potential pathogenic taxa before and after treatment with iron, GOS, and bLF in PolyFermS. Mean ± SD of log_10_ bacteria/mL effluent is shown for the last three days of stabilization (STAB) and the last three days of treatment (TREAT) of (A) two experimental periods of donor microbiota 4 and (B) three experimental periods of donor microbiota 5. Significant differences between STAB and TREAT in the corresponding periods are indicated. ^*^P < 0.05, ^**^P < 0.01. *Enterobacteriaceae* and *C. difficile* were also detected in donor 5 [Supplementary Figure 5]. GOS: Galacto-oligosaccharides; bLF: bovine lactoferrin.

In line with batch experiments, supplementation of GOS during iron treatment stimulated the growth of beneficial bacteria during continuous cultivation [Figure 3]. Iron with GOS alone and combined with bLF resulted in a similar increase in *Bifidobacterium* at the end of all treatment periods compared to the levels at stabilization for donor 4 (average increase of 0.58 log/mL for GOS and 0.53 log/mL for GOS + bLF) and donor 5 microbiota (average increase of 0.70 log/mL for GOS and 0.64 log/mL for GOS + bLF). LLP showed an increase only in donor 5 microbiota upon GOS (average increase of 0.54 log/mL) and GOS + bLF (average increase of 0.52 log/mL) treatment during iron supplementation compared to stabilization levels. A similar decrease in potentially pathogenic bacteria was detected in donor 4 microbiota with GOS and GOS + bLF for *Enterobacteriaceae* (average decrease of -0.61 and -0.70 log/mL, respectively) and *C. difficile* (average decrease of -0.80 and -1.16 log/mL, respectively). Additionally, *Clostridium perfringens* decreased during treatment period 1 (-1.33 log/mL for GOS and -1.60 log/mL for GOS + bLF), but not during period 2 in donor 4 microbiota. Supplementation with iron alone and iron with bLF did not result in major repeatable changes in bacterial concentrations during treatment compared to the non-supplemental control. In donor 5 microbiota, however, *Bifidobacterium* decreased during treatment period 1 with iron supplementation (-0.14 log/mL) and *C. difficile* increased in two of three treatment periods with iron (+0.24 and +1.13 log/mL) and iron + bLF (+0.25 and +0.82 log/mL) supplementation compared to the non-supplemented control (+0.15 and +0.51 log/mL) [Supplementary Figure 5]. Total bacterial counts were not different between stabilization and treatment periods [Supplementary Figure 5].

**Figure 4:**
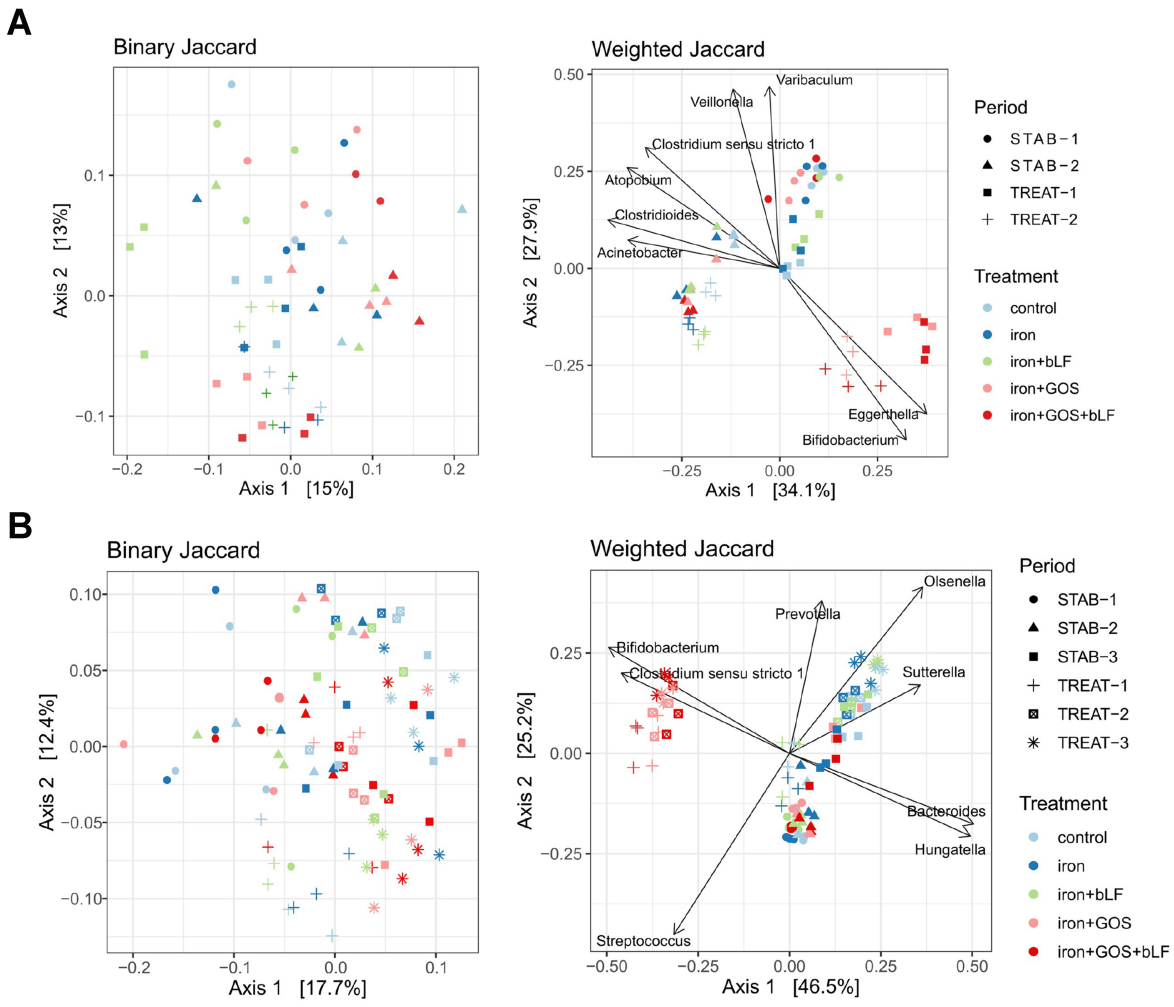
PCoA of binary and weighted Jaccard distance metrics of before and after treatment with iron, GOS and bLF in PolyFermS. The last three days of stabilization and treatment of two and three experimental periods are shown for (A) donors 4 and (B) 5, respectively. The top 8 genera associated with the community composition are plotted as vectors in weighted Jaccard. PCoA: Principal coordinate analysis; GOS: galacto-oligosaccharides; bLF: bovine lactoferrin.

Treatment effects on community composition and structure were assessed by 16S rRNA gene amplicon sequencing. Principal coordinate analysis (PCoA) of weighted Jaccard distance revealed distinct clustering of microbiota treated with GOS alone and GOS + bLF during iron supplementation in donor 4 (driven by *Bifidobacterium* and *Eggerthella*, Figure 4A) and donor 5 microbiota (driven by *Bifidobacterium* and *Clostridium* sensu stricto 1, Figure 4B). Compared with iron supplementation alone, significant shifts in weighted Jaccard distance were detected for treatments with GOS alone and combined with bLF in donor 4 [Supplementary Figure 6B] and 5 [Supplementary Figure 7B] microbiota.

**Figure 5:**
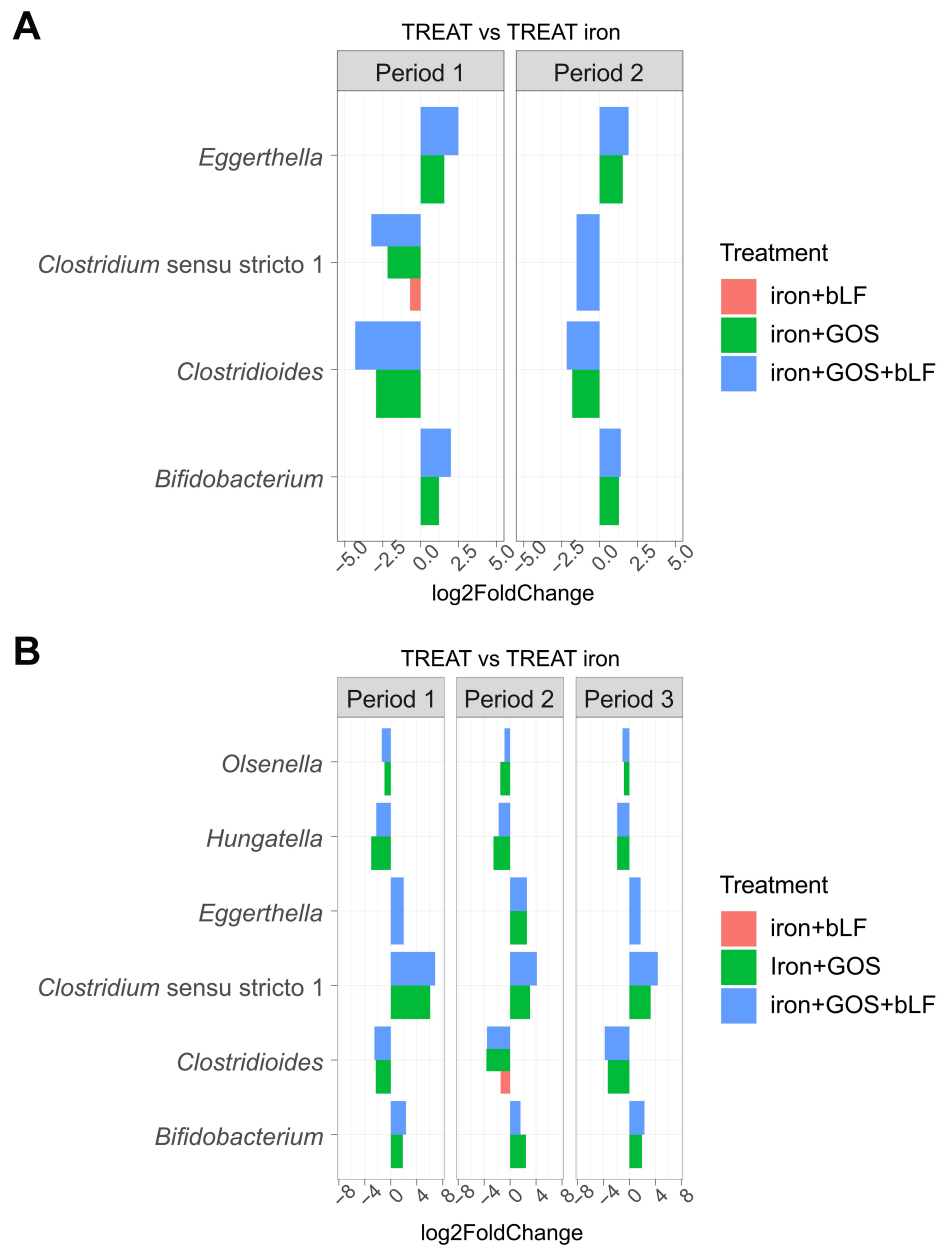
Differential abundance analysis (DESeq2) at genus level after treatment with iron and GOS with/without bLF in PolyFermS. Barplots show log_2_-fold changes of genera significantly (*P* < 0.05) different in relative abundance between the last three days of iron co-supplementation with GOS and/or bLF and the last three days of supplementation with iron alone for all experimental periods in (A) donor 4 and (B) donor 5 microbiota. GOS: Galacto-oligosaccharides; bLF: bovine lactoferrin.

Differential abundance analysis with DESeq2 detected several genera that were significantly different in relative abundance during the last three days of treatment compared to the last three days of stabilization [Supplementary Figure 8]. In addition, when comparing the last three treatment days of each iron co-supplementation strategy to the last days of iron supplementation alone, several of these genera were significantly different in relative abundance [Figure 5].

Co-supplementation of iron + GOS, alone or combined with bLF, resulted in consistent changes in both tested microbiota with increased relative abundance of *Bifidobacterium* and *Eggerthella* and decreased *Clostridioides* (assigned to *C. difficile*, up to -4.3 log2FoldChange) [Figure 5]. Further, the relative abundance of genus *Clostridium* sensu stricto decreased in donor 4 (all ASVs were assigned to *C. perfringens*) while it increased in donor 5 (all ASVs were assigned to *C. neonatale*) during iron + GOS and iron + GOS + bLF supplementation compared to iron alone supplementation [Figure 5].

No consistent differences in genus relative abundance were detected when comparing the microbiota treated with iron + GOS to those treated with iron + GOS + bLF. Further, the microbiota community evenness decreased for donor 4 microbiota after iron + GOS and iron + GOS + bLF treatment compared to the stabilization period, likely associated with the promotion of the dominant *Bifidobacterium* leading to a more uneven community [Supplementary Figure 9].

**Figure 6:**
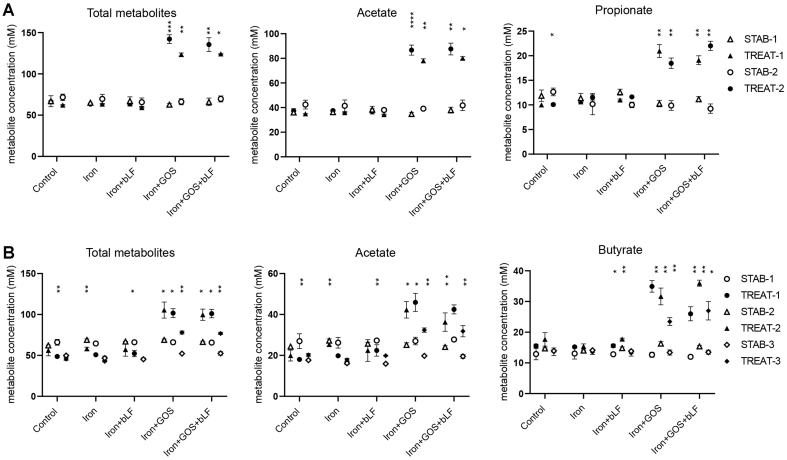
Quantification of total and intermediate metabolites and SCFA after treatment with iron and GOS with/without bLF in PolyFermS. Mean ± SD of metabolite concentration is shown for the last three days of stabilization (STAB) and the last three days of treatment (TREAT) of (A) two experimental periods of donor 4 and (B) three experimental periods of donor 5. Significant differences between STAB and TREAT in the corresponding periods are indicated. ^*^P < 0.05, ^**^P < 0.01, ^***^P < 0.001, ^****^P < 0.0001. SCFA: Short-chain fatty acid; GOS: galacto-oligosaccharides; bLF: bovine lactoferrin.

As expected, total metabolite production was increased upon iron + GOS (+58 to 76 mM) and iron + GOS + bLF (+24 to 36 mM) supplementation in both donor microbiota compared to the stabilization period concentrations [Figure 6 and Supplementary Figure 10]. Production of acetate in both donors and propionate in donor 4 and butyrate in donor 5 microbiota was enhanced in treatments containing GOS compared to stabilization.

In summary, no effect of iron supplementation alone was observed on microbiota composition and metabolic activity. The addition of GOS alone and combined with bLF during iron supplementation promoted SCFA production and beneficial taxa during continuous cultivations in PolyFermS and decreased potential harmful bacteria, including *C. difficile* and *C. perfringens*.

### GOS and bLF-treated microbiota supernatants strengthen the epithelial barrier and protect from infection-induced effects donor-dependently

Finally, it was assessed whether the fermentation supernatants from treated PolyFermS microbiota with iron, iron + bLF, iron + GOS, and iron + GOS + bLF affected the epithelial barrier, infection, and inflammatory response during ETEC infection differently from the non-treated PolyFermS microbiota in an *in vitro* model of epithelial and immune cells.

**Figure 7:**
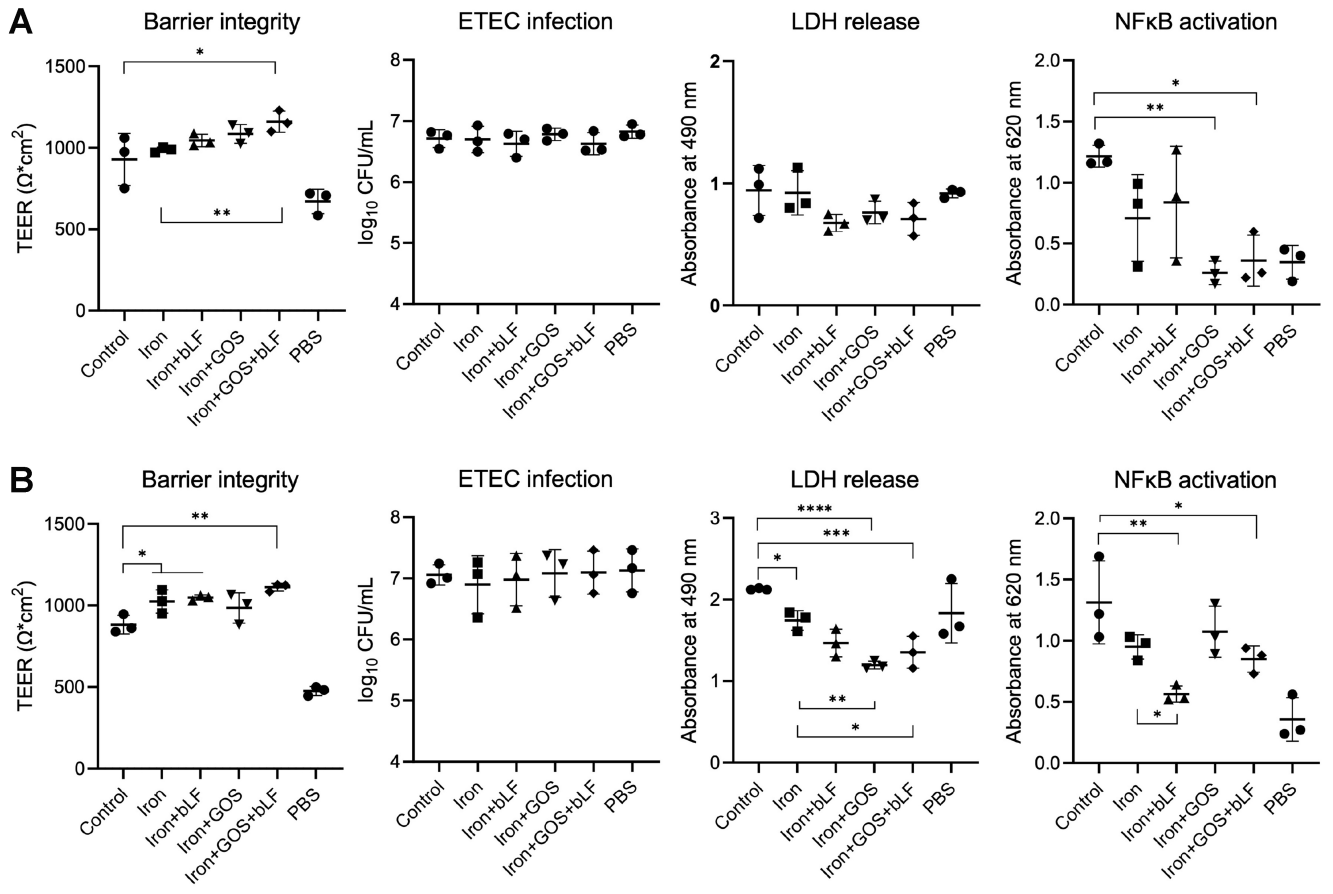
Effect of PolyFermS supernatant treatments on epithelial barrier, infection and inflammation in a Caco-2/HT29-MTX/THP-1 Blue cell co-culture model. Barrier integrity was assessed by TEER measurement after 24-h exposure to 20% of microbiota supernatant of (A) donor 4 and (B) donor 5. CFU of adhered and invaded ETEC and cell cytotoxicity were assessed by plating and LDH release, respectively, after 3 h of infection in the presence of 20% microbiota supernatant. NFκB activation in THP1-Blue cells was assessed after 21 h of further incubation following infection. Mean ± SD is shown, *n* = 3 independent cell passages. TEER: Transepithelial electrical resistance; CFU: colony forming units; ETEC: enterotoxigenic *Escherichia coli*; LDH: lactate dehydrogenase; NFκB: nuclear factor kappa B.

The epithelial barrier was strengthened when all types of PolyFermS supernatants were added, as indicated by higher TEER values compared to the negative control PBS [Figure 7]. The increase in TEER was higher with the supernatant from iron + GOS + bLF compared to iron-alone treated microbiota of donor 4 (1,160 *vs.* 989 Ω·cm^2^) [Figure 7A]. ETEC adhesion and invasion were not impacted by the addition of any of the microbiota supernatants [Figure 7] or direct iron supplementation at 5 mg/L [Supplementary Figure 11]. Infection-induced cell death (assessed by LDH release) was only decreased by supernatant of iron + GOS- and iron + GOS + bLF-treated donor 5 microbiota compared to iron-treated microbiota [Figure 7B]. The infection-induced pro-inflammatory response was stimulated when the control microbiota supernatant was added, with a 3.6-fold increased NFκB activation in control compared to PBS [Figure 7]. In contrast, the addition of iron + GOS- and iron + GOS + bLF-treated supernatant of donor 4 microbiota did not result in increased NFκB activation [Figure 7A]. Iron + bLF supernatant of donor 5 microbiota significantly reduced the pro-inflammatory response compared to iron alone supernatant [Figure 7B]. TEER values obtained with donor 4 supernatants were negatively correlated with the relative abundance of *Finegoldia* (Spearman r = -1.0, *P* = 0.017), and NFκB activation was positively correlated with the relative abundance of *Pseudomonas* (Spearman r = 1.0, *P* = 0.017), although the relative abundance of both genera was below 1% [Supplementary Table 7]. The LDH release with donor 5 supernatant was negatively correlated with acetate and butyrate (Spearman r = -1.0, *P* = 0.017) and positively correlated with *Bacteroides*, *Hungatella*, *Flavonifractor*, and *Lachnoclostridium* (Spearman r = 1.0, *P* = 0.017).

In summary, a strengthening of the epithelial barrier and a decrease in cell death and pro-inflammatory response during ETEC infection was observed with microbiota fermentation supernatants from iron + GOS, iron + bLF, and iron + GOS + bLF treatments compared to iron alone.

## DISCUSSION

In this study, we investigated the effect of low-dose iron supplementation, alone and combined with bLF, GOS, and GOS + bLF, on the gut microbiota of infants living in a rural area of Kenya and at the age of weaning (5.6-9.7 months old) in the recently validated Kenyan infant PolyFermS microbiota model^[26]^.

Iron supplementation mimicking 5 mg of iron/day did not induce substantial changes in the five investigated Kenyan infant gut microbiota during batch and continuous fermentations *in vitro*. This agrees with our previous Kenyan infant PolyFermS study^[27]^, where iron supplementation mimicking a higher dose of 12.5 mg iron/day also did not induce major changes in four investigated Kenyan infant gut microbiota, except for an increase in *C. difficile* abundance in one microbiota as observed here in donor 5 microbiota as well. Other *in vitro* studies also reported none^[43]^ or minimal^[44]^ changes in human gut microbiota composition with iron supplementation. These *in vitro* studies are in contrast to the *in vivo* observed increase in *Enterobacteriaceae* or enteropathogens and decrease in *Bifidobacterium* in the fecal microbiota of 6.0-9.5-month-old Kenyan infants after iron fortification (5 and 12.5 mg iron/day)^[3,8,9]^. This suggests that the adverse effects of iron on the fecal microbiota reported *in vivo* may be due to not yet identified iron-host-microbiota interactions in specific individuals rather than being a result of direct iron-gut microbiota interactions.

The addition of GOS alone and combined with bLF during iron supplementation promoted SCFA production and growth of beneficial *Bifidobacterium* and LLP, while a decrease in potentially harmful bacteria, including *C. difficile* and *C. perfringens* was observed. This observation agrees with our previous studies where increased *Bifidobacterium* and/or *Lactobacillus* relative abundance concomitant with the decreased enteropathogen markers was observed in the microbiota of weaning 6.0-9.5-month-old Kenyan infants treated with GOS^[3]^ or with a mixture of short-chain GOS (90%) and long-chain FOS (10%) during iron supplementation *in vitro*^[27]^ or *in vivo*^[11]^. The bifidogenic and metabolic effects of GOS have been reported in cohort studies and *in vitro* fermentation studies with Western infants aged 4 to 24 months^[45-50]^. Growth inhibition of GOS on enteropathogens in our study may be explained by the production of bacteriocins by GOS-promoted bifidobacteria^[51,52]^ and by the higher SCFA levels, as SCFA were inversely correlated with *C. difficile* pathogenesis *in vivo*^[53,54]^ and addition of acetate, propionate, and butyrate (40-100 mM) inhibited growth of *C. difficile* in culture by 75%^[55]^. With pH controlled in our *in vitro* systems, we expect a stronger SCFA-induced pathogen reduction in Kenyan infants treated with GOS and GOS + bLF during iron supplementation than observed *in vitro*.

The addition of bLF during iron supplementation did not impact the composition and metabolite profile of Kenyan infant gut microbiota in our *in vitro* study. Previous clinical studies in young pre-weaning infants reported an effect of bLF supplementation, with an increase in *Bacteroides* and a reduction in *Enterobacter*, *Klebsiella*, *Staphylococcus*, *Haemophilus*, and *Lactobacillus* compared to control groups^[56-58]^. In contrast, a recent study found no impact of bLF on the fecal microbiota and metabolome of 8-month-old infants^[59]^. The antimicrobial activity of bLF against enteropathogens, such as pathogenic *E. coli* and *Clostridium* spp., was observed in mice and in pure culture experiments^[60-63]^. In our study, the lack of antimicrobial effect of apo-bLF (< 5% iron saturated) on *C. difficile* and *C. perfringens* may be partly explained by the low tested dose of 1.1 mg/mL that mimicked the daily dose of 1 g bLF in the cohort trial^[20]^. It was previously reported that growth inhibition of *C. difficile* and *C. perfringens* by bLF (15%-20% iron saturated) occurred at a minimal concentration of 16 mg/mL^[61]^. Another possible explanation for the lack of pathogen inhibition in our experiments might be the form in which bLF was supplemented in our model. In an *in vitro* gut microbiota model for *C. difficile* infection, 5 mg/mL of apo-bLF did not affect *C. difficile* growth and toxin production while holo-bLF (85% iron saturated) led to inhibition of both^[64]^.

Several studies reported iron-induced adhesion of opportunistic enteropathogens, such as *E. coli* and *Salmonella*, to epithelial cells *in vitro*^[25,65,66]^. Adhesion of ETEC to HT29 cells doubled when a high dose of 100 µM iron was added but not with a lower iron dose of 50 µM, compared to the control^[66]^. This iron dose effect on ETEC adhesion may explain our results, as in our ETEC infection study of Caco-2/HT29-MTX cells, iron concentration in the supplemented fermentation supernatants were low with 0.4 mg/L (0.7 µM) and 0.6 mg/L (1.1 µM) for donor 4 and 5, respectively. Direct iron supplementation of cell medium at 5 mg/L (9 µM) to mimic the daily dose of 5 mg iron in the cohort trial^[20]^ also did not impact ETEC infection. Interestingly, infection-induced cell death was decreased by supernatant from donor 5 microbiota treated with bLF and GOS during iron supplementation and this was correlated with increased concentrations of acetate and butyrate in those supernatant samples. Butyrate was previously shown to suppress Caco-2 cell death^[67]^, while acetate and butyrate are energy substrates for epithelial cells^[68]^.

One limitation of this study is that the 16S rRNA gene short-amplicon sequencing approach does not provide taxonomic species- or strain-level resolution. Employing metagenomic sequencing could overcome this limitation, offering detailed insights into whether the predominant fecal *Bifidobacterium* strains were retained *in vitro*. Moreover, metagenomic data could elucidate potential cross-feeding interactions in response to GOS-containing treatments, thereby enhancing our mechanistic understanding of these treatments. Another study limitation is that with the used *in vitro* models, we could not observe an iron-induced microbial dysbiosis as previously reported *in vivo*. *In vitro* gut microbiota modeling does not account for all host- and environmental-related factors, and the conditions of the fermentations were tightly set^[26]^. Combining different microbiota models (*in vitro*, *ex vivo*, *in silico*, and animal) and integrating them with cohort data may help overcome this limitation when studying complex microbial ecosystems in disease^[22]^.

In conclusion, this *in vitro* study is the first to evaluate the impact of a novel combination of GOS with bLF on Kenyan infant microbiota during low-dose iron supplementation. Using the Kenyan infant PolyFermS microbiota model, we demonstrated that supplementation with GOS alone or combined with bLF elicited bifidogenic effects, inhibited enteropathogens, and promoted SCFA production, while bLF and iron alone showed no significant impact. Additionally, combining *in vitro*-treated microbiota with a mammalian cell model identified donor-dependent beneficial effects of GOS and bLF on the epithelial barrier and immune response. The results from this *in vitro* study will aid in interpreting outcomes from a parallel cohort study in the same infant population, advancing our understanding of the potential benefits of GOS combined with bLF in enhancing infant gut health during iron supplementation.

## DECLARATIONS

### Availability of data and materials

The datasets generated and/or analyzed during the current study are available in the European Nucleotide Archive (ENA) repository, accession number PRJEB63476 (https://www.ebi.ac.uk/ena/browser/view/ PRJEB63476). Other raw data that support the findings of this study are available from the corresponding author upon reasonable request.

### Copyright

© The Author(s) 2024.
