# Supplementary material for: Galacto-oligosaccharides alone and combined with lactoferrin impact the Kenyan infant gut microbiota and epithelial barrier integrity during iron supplementation in vitro
Source: Microbiome Res Rep. 2024 Dec 17;4(1):9. doi: 10.20517/mrr.2024.34 (PMC11978484; doi:10.20517/mrr.2024.34)
Supplement: Supplementary file 1 [file mrr-4-1-9-SupplementaryMaterials.pdf]

## Supplementary Materials

**Galacto-oligosaccharides alone and combined with lactoferrin impact the Kenyan infant gut microbiota and epithelial barrier integrity during iron supplementation *in vitro***

**Carole Rachmühl[1], Christophe Lacroix[1], Adele Ferragamo[1,2], Ambra Giorgetti[3], Nicole U. Stoffel[3], Michael B. Zimmermann[4], Gary M. Brittenham[5], Annelies Geirnaert[1]**

[1]Laboratory of Food Biotechnology, Institute of Food, Nutrition and Health, Department of Health Sciences and Technology, ETH Zürich, Zürich 8092, Switzerland.

[2]Current address: Clean Mouse Facility, Experimental Animal Center, Department for Biomedical Research, University of Bern, Bern 3008, Switzerland.

[3]Laboratory of Human Nutrition, Institute of Food, Nutrition and Health, Department of Health Sciences and Technology, ETH Zürich, Zürich 8092, Switzerland.

[4]Medical Research Council Translational Immune Discovery Unit, Weatherall Institute of Molecular Medicine, John Radcliffe Hospital, University of Oxford, Oxford OX3 9DS, UK.

[5]Department of Pediatrics, College of Physicians and Surgeons, Columbia University, New York, NY 10032, USA.

**Correspondence to:** Prof. Christophe Lacroix, Dr. Annelies Geirnaert, Laboratory of Food Biotechnology, Institute of Food, Nutrition and Health, Department of Health Sciences and Technology, ETH Zürich, Schmelzbergstrasse 7, Zürich 8092, Switzerland. E-mail: christophe.lacroix@hest.ethz.ch; annelies.geirnaert@hest.ethz.ch

**Supplementary methods**

**Supplementary Table 1**: Characteristics of the fecal donors and fecal pH measurement.

| Donor | Sex | Age (months) | Diet | Time since solid food introduction (months) | Delivery | Gestation at birth | Birth weight (kg) | Fecal pH |
|---|---|---|---|---|---|---|---|---|
| 1 | Male | 9.7 | Breast milk (8x/day), porridge, potatoes, vegetables, fruits | 4.7 | Vaginal | Term | 2.0 | 7.3 |
| 2 | Female | 8.2 | Breast milk (10x/day), porridge, potatoes, vegetables, fruits | 2.2 | Vaginal | Term | 2.0 | 4.8 |
| 3 | Male | 7.6 | Breast milk (4x/day), porridge, fruits, vegetables | 1.6 | Vaginal | Term | 3.4 | 4.3 |
| 4 | Male | 5.6 | Breast milk (>10x/day) | 0.0 | Vaginal | Term | 2.8 | 4.8 |
| 5 | Female | 5.6 | Breast milk (>10x/day) | 0.0 | Vaginal | Term | 3.1 | 5.3 |

**Supplementary table 2**. Composition of the fermentation medium is designed to mimic the ileal chyme entering the proximal colon of Kenyan infants during weaning.

| **Component** (supplier) | **g/L of distilled water** | |
|---|---|---|
| | **Continuous (PolyFermS)** | **Batch (24 well plate)** |
| Zein (47% of total protein) (Sigma-Aldrich, Buchs, Switzerland) | 0.3 | 0.3 |
| Gluten hydrolysate from maize (39% of total protein) (Neolab, Heidelberg, Germany) | 0.3 | 0.3 |
| Corn starch (Sigma-Aldrich) | 0.3 | 0.15 |
| Xylan (beechwood) (Chemie Brunschwig AG, Basel, Switzerland) | 0.4 | 0.2 |
| Arabinogalactan (larch wood) (Lonza, Basel, Switzerland) | 2.2 | 1.1 |
| Fructo-oligosaccharides (short-chain) (Fibrulose F97, Cosucra, Pecq, Belgium) | 1.0 | 1.0 |
| D-lactose (Sigma-Aldrich) | 3.2 | 1.6 |
| Casein hydrolysate (Sigma-Aldrich) | 0.3 | 0.3 |
| Whey protein hydrolysate (Emmi, -Dagmarsellen, Switzerland) | 4.1 | 4.1 |
| Peptone from casein (Sigma-Aldrich) | 0.5 | 0.5 |
| Bacto™ Tryptone (Chemie Brunschwig AG) | 0.5 | 0.5 |
| Mucin (Sigma-Aldrich) | 4.0 | 4.0 |
| Yeast extract (Standard nucleotide yeast extract) (VWR International AG, Dietikon, Switzerland) | 2.5 | 2.5 |
| L-cysteine HCl monohydrate (Sigma-Aldrich) | 0.8 | 0.8 |
| Bile salts (Thermo Fisher, Pratteln, Switzerland) | 0.05 | 0.05 |
| $KH_2PO_4$ (VWR International AG) | 0.5 | - |
| $NaHCO_3$ (Sigma-Aldrich) | 1.5 | 9 |
| $C_6H_{13}NO_4S$ (MES) (VWR International AG) | - | 4.3 |
| NaCl (VWR International AG) | 4.5 | 4.5 |
| KCl (Sigma-Aldrich) | 4.5 | 4.5 |
| $MgSO_4$ Anhydrous (Sigma-Aldrich) | 1.3 | 1.3 |
| $CaCl_2 \cdot 2H_2O$ (Sigma-Aldrich) | 0.1 | 0.1 |
| Hemin (Sigma-Aldrich) | 0.01 | 0.01 |
| Tween 80 (Sigma-Aldrich) | 1.0 mL | 1.0 mL |
| Vitamin solution | 0.5 mL | 0.5 mL |
| The composition of the vitamin solution was (mg/L of distilled water): thiamine-HCl (Vit B1-HCl;50), (-)-riboflavin (Vit B2; 50), nicotinic acid (Vit B3; 50), pantothenic acid (Vit B5; 100), pyridoxine-HCl (Vit B6; 100), folic acid (Vit B9, 20), cyanocobalamin (Vit B12; 5), 4-aminobenzoic acid (PABA; 50), biotine (Vit H; 20), phylloquinone (Vit K1; 0.075), and menadione (Vit K3; 10). (All purchased from Sigma-Aldrich) | | |

*Cultivation medium preparation*

The pH of the medium for batch cultivation was adjusted to 7.0 using 2.5 M NaOH prior to heating until boiling using a heating plate (IKA, Staufen, Germany) and an Allihn condenser (Lenz Laborglas GmbH, Wertheim, Germany). Subsequently, the medium was flushed with $CO_2$ for 15 min and filled

into $CO_2$-flushed serum flasks, which were closed with butyl rubber and sealed with an aluminum cap. The serum flasks were autoclaved at 121 °C for 20 min. The medium used for continuous fermentation was prepared aerobically and the pH was adjusted to 5.8 using 5 M HCl prior to autoclaving at 121 °C for 20 min. The filter-sterilized vitamin and FOS solutions were added after autoclaving of both media.

*Fecal microbiota immobilization and PolyFermS bioreactor operation*

The fecal microbiota was immobilized into porous gel beads (2.5% (w/v) gellan gum, 0.25% (w/v) xanthan gum, 0.2% (w/v) sodium citrate) using a two-phase dispersion process in an anaerobic chamber [1]. Fecal beads were inoculated into the inoculum bioreactor (IR) (Multifors system, Infors AG, Switzerland and DASbox system, Vaudaux-Eppendorf AG, Basel, Switzerland) containing 140 mL (30%, v/v) of cultivation medium. To colonize the beads, two successive batch fermentations were carried out at 37 °C, pH 5.8 controlled by the addition of 2.5 M NaOH (EasyFerm Plus-PHI Arc sensors, Hamilton, Bonaduz, Switzerland), stirring at 120 rpm and by replacing 100 mL of fermented effluent with fresh medium after 20 and 6 h, respectively. The pH of IR containing fecal microbiota beads of donors 1, 2, 4, and 5 was set at 5.8, whereas donor 3 IR pH was operated at 6.3. Subsequently, the operation was switched to continuous mode with inflow of fresh cultivation medium (25 mL/h) and outflow of fermented effluent at the same rate for a total volume of 200 mL and a retention time of 8 h. Anaerobiosis was ensured by flushing the reactor headspace with filter-sterile $CO_2$ and monitored by redox potential probes (EasyFerm Plus-ORP Arc sensors, Hamilton, Bonaduz, Switzerland).

*Cell lines and culture conditions*

Caco-2 intestinal adsorptive cells (DSMZ ACC 169, passages 14 to 37) and mucus-secreting HT29-MTX cells (ECACC 12040401, passages 67 to 83) were routinely maintained in Dulbecco's Modified Eagle medium (DMEM) Glutamax supplemented with 10% fetal bovine serum (FBS), 1% penicillin/streptomycin, and 1% non-essential amino acids (NEAA) (all Thermo Fisher Scientific). Human peripheral blood monocytic THP-1 Blue cells (Invivogen, thp-nfkb) were routinely maintained in RPMI 1640 supplemented with 10% heat-inactivated FBS, 1% penicillin/streptomycin, 1x NEAA, 1x MEM vitamin solution, 2 mM L-glutamine, 1 mM Na-pyruvate (all Thermo Fisher Scientific), and 100 µg/mL zeocin (Invivogen, LabForce AG, Muttenz, Switzerland). THP1-Blue cells were transfected with an NFκB-inducible secreted embryonic alkaline phosphatase (SEAP) reporter construct (Invivogen), which enables the screening of pro-inflammatory NFκB activation. All cell lines were cultivated at 37 °C in a humified incubator (5% $CO_2$, MultiTemp Scientific AG, Kloten, Switzerland).

*Ferrozine assay to estimate the iron concentration in PolyFermS culture supernatant*

The ferrozine assay was used for colorimetric quantification of total elemental iron as previously described [2,3]. In brief, 360 µL (31% v/v) of the samples were mixed with 800 µL of trichloroacetic acid solution (20% w/v in $dH_2O$) solution, boiled for 10 min at 100 °C and subsequently centrifuged at

12000 g for 5 min to pellet debris. The supernatant was mixed with a ferrozine stock solution (17 mM sodium acetate, 4.6 mM L-sodium ascorbate, and 0.18 mM 3-(2-pyridyl)-5,6-diphenyl-1,2,4-triazine-p,p'-disulfonic acid monosodium salt hydrate in $dH_2O$, all Sigma-Aldrich Chemie AG, Buchs, Switzerland) at 25% v/v and the absorbance was measured at 560 nm using a spectrophotometer (Biotek microplate reader, Agilent, Santa Clara, CA, USA), in triplicate. A commercial $FeCl_3$ solution (Titrisol 1000 mg Fe/L, Sigma-Aldrich Chemie GmbH, Buchs, Switzerland) was used to generate the standard curve with serial-fold dilutions.

**Supplementary Table 3: Primers used for PCR and qPCR analysis for fecal and fermentation samples**

| Target | Primers | 5'-3' sequence | Target gene | Amplicon length [bp] | Reference |
|---|---|---|---|---|---|
| Total Bacteria | Eub338F | ACT CCT ACG GGA GGC AGC AG | 16S rRNA gene | 200 | [4] |
| | Eub518R | ATT ACC GCG GCT GCT GG | | | |
| *Enterobacteriaceae* | Eco1457F | CAT TGA CGT TAC CCG CAG AAG AAG C | 16S rRNA gene | 195 | [5] |
| | Eco1652R | CTC TAC GAG ACT CAA GCT TGC | | | |
| *Lactobacillus*/*Pediococcus* /*Leuconostoc* (LLP) | F_Lacto 05 | AGC AGT AGG GAA TCT TCC A | 16S rRNA gene | 375 | [6] |
| | R_Lacto 04 | CGC CAC TGG TGT TCY TCC ATA TA | | | |
| *Bifidobacterium* | Bif F | TCG CGT CYG GTG TGA AAG | 16S rRNA gene | 243 | [7] |
| | Bif R | CCA CAT CCA GCR TCC AC | | | |
| *Clostridioides difficile* | cdF | TTG AGC GAT TTA CTT CGG TAA AGA | 16S rRNA gene | 157 | [7] |
| | cdR | CCA TCC TGT ACT GGC TCA CCT | | | |
| Enteropathogenic *Escherichia coli* (EPEC) | EAE-a | ATG CTT AGT GCT GGT TTA GG | eaeA (E. coli attaching and effacing) | 248 | [8] |
| *Clostridium perfringens* | plcF | AAG TTA CCT TTG CTG CAT AAT CCC | plc (alpha toxin) | 283 | [7] |
| | plcR | ATA GAT ACT CCA TAT CAT CCT GCT | | | |

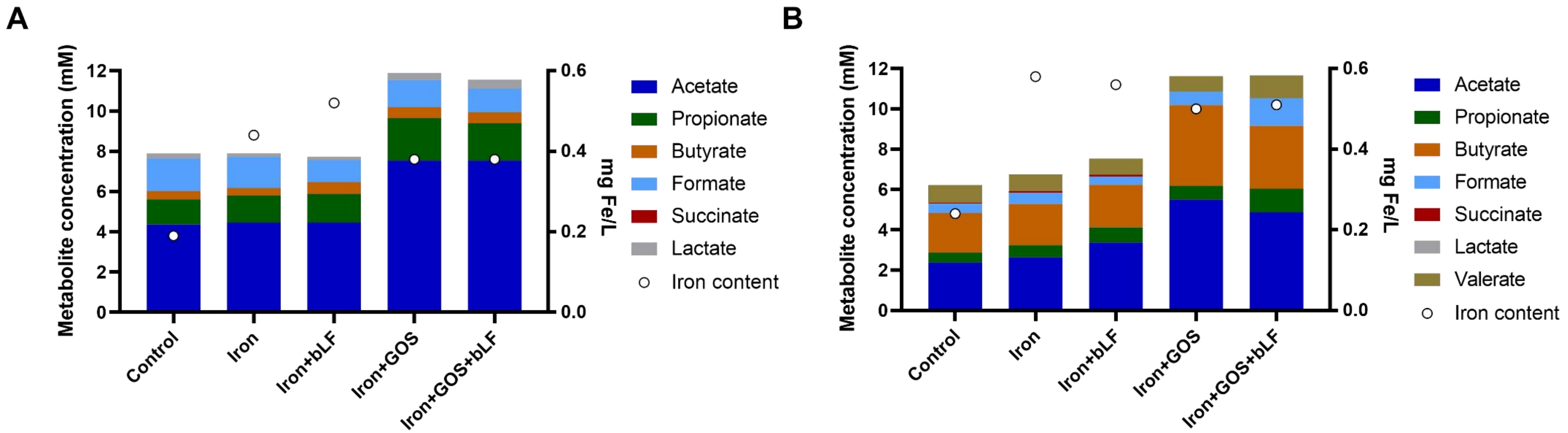


**Supplementary figure 1**. Metabolite profile and iron content of PolyFermS culture supernatant from donor 4 (**A**) and donor 5 (**B**) used for Caco-2/HT29-MTX/THP-1 co-culture experiments. Intermediate metabolite and SCFA concentrations (left y-axis) and concentration of elemental iron (right y-axis) that were applied to the mammalian cells.

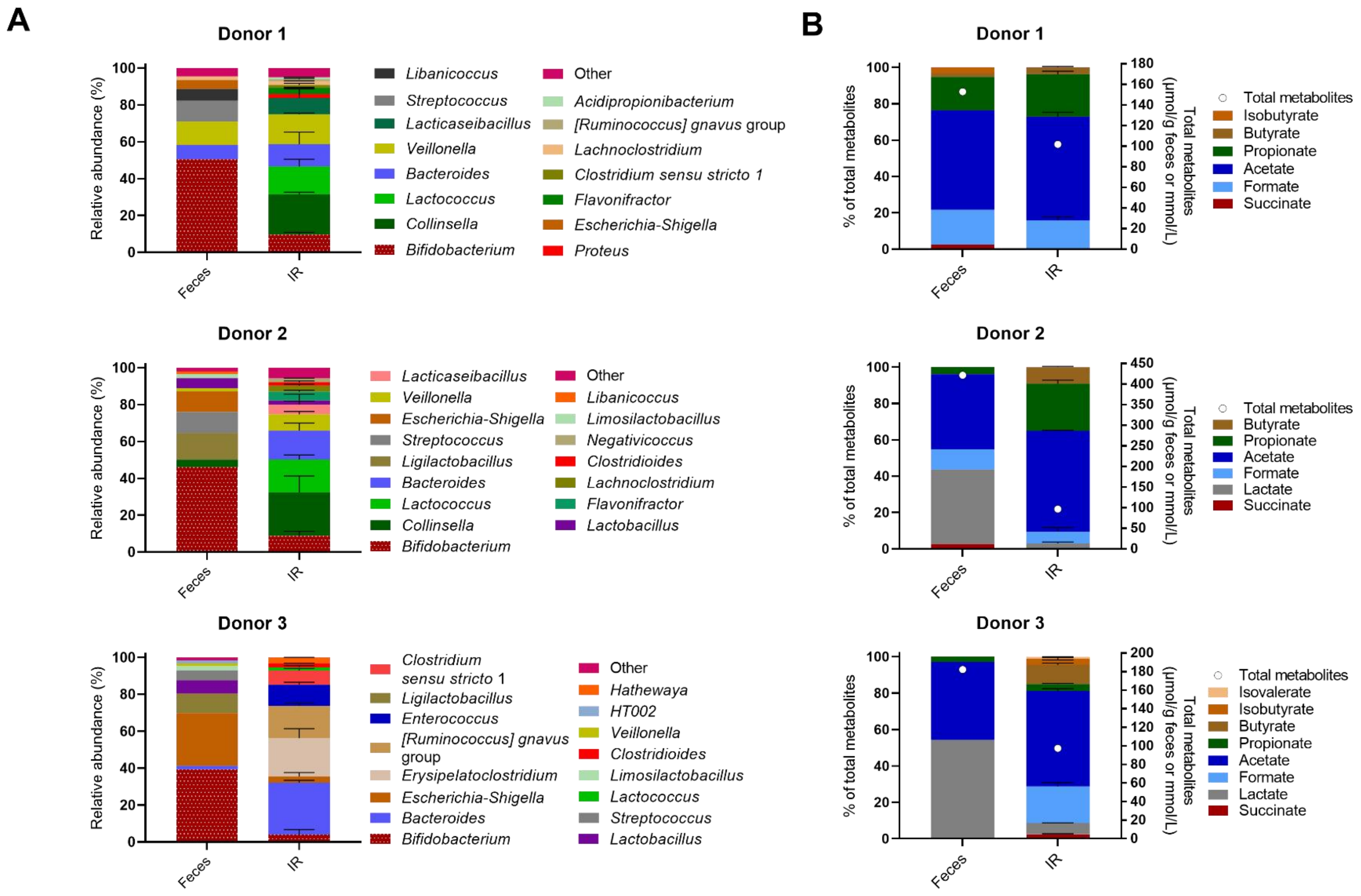


**Supplementary figure 2.** Microbiota composition and metabolite profile of donor 1, 2 and 3 feces and corresponding PolyFermS microbiota that were used as inoculum for 24 h batch fermentation. (**A**) Relative abundance on genus level («other»: <1%). (**B**) Proportions of SCFA, BCFA and intermediate fermentation metabolites (left y-axis) and total metabolite concentrations (right y-axis, circles). Mean ± SD of fermentation on days 114-116, days 107-109 and days 39-41 are shown for the inoculum reactor (IR) sample of donors 1, 2 and 3, respectively.

**Supplementary table 4.** Fermented sample pH for treatment with iron, GOS and bLF after 24 h batch fermentation.

| | Donor 1 | Donor 2 | Donor 3 |
|---|---|---|---|
| Start pH | 7.2 ± 0.1 | 7.0 ± 0.1 | 7.1 |
| Control | 7.4 ± 0.2 | 7.2 ± 0.1 | 7.4 ± 0.2 |
| Iron 0.5x | 7.2 ± 0.1 | 7.1 ± 0.1 | 7.3 ± 0.2 |
| Iron 1x | 7.2 ± 0.1 | 7.2 ± 0.1 | 7.4 ± 0.2 |
| Iron 2x | 7.4 ± 0.2 | 7.3 ± 0.0 | 7.5 ± 0.3 |
| Iron 1x bLF 0.5x | 7.2 ± 0.1 | 7.1 ± 0.1 | 7.4 ± 0.2 |
| Iron 1x bLF 1x | 7.2 ± 0.1 | 7.1 ± 0.1 | 7.3 ± 0.2 |
| Iron 1x bLF 2x | 7.3 ± 0.2 | 7.2 ± 0.1 | 7.4 ± 0.1 |
| Iron 1x GOS 0.12x | 7.1 ± 0.1 | 6.8 ± 0.2 | 6.9 ± 0.5 |
| Iron 1x GOS 0.25x | 6.6 ± 0.1 | 6.2 ± 0.2 | 6.4 ± 0.5 |
| Iron 1x GOS 0.5x | 6.0 ± 0.1 | 5.4 ± 0.2 | 5.5 ± 0.3 |
| Iron 1x bLF 0.5x GOS 0.12x | 7.0 ± 0.1 | 6.8 ± 0.1 | 6.9 ± 0.4 |
| Iron 1x bLF 1x GOS 0.25x | 6.6 ± 0.1 | 6.2 ± 0.3 | 6.3 ± 0.4 |
| Iron 1x bLF 2x GOS 0.5x | 6.0 ± 0.2 | 5.5 ± 0.2 | 5.7 ± 0.3 |

Mean ± SD is shown (except for start pH donor 3). n = 3 repeats per donor with inoculum derived from three consecutive fermentation days.

**Supplementary Table 5.** Quantification of key bacterial taxa in infant microbiota treated with iron, GOS and bLF during 24 h batch fermentations in 24-well plates.

| | | Total bacteria (16S copies/mL) | | | *Enterobacteriaceae* (bacteria/mL) | | |
|---|---|---|---|---|---|---|---|
| | | Donor 1 | Donor 2 | Donor 3 | Donor 1 | Donor 2 | Donor 3 |
| $log_{10}$ | Inoculum | 10.60 ± 0.14 | 10.29 ± 0.14 | ND | 7.02 ± 0.55 | 6.62 ± 0.19 | ND |
| | Control | 10.78 ± 0.15 | 10.88 ± 0.04 | 10.76 ± 0.11 | 8.73 ± 0.47 | 9.09 ± 0.09 | 8.56 ± 0.12 |
| $\Delta log_{10}$ compared to control | Iron 0.5x | -0.01 ± 0.09 | 0.09 ± 0.26 | -0.07 ± 0.07 | 0.33 ± 0.15 | 0.16 ± 0.23 | -0.09 ± 0.08 |
| | Iron 1x | 0.07 ± 0.12 | -0.04 ± 0.02 | -0.04 ± 0.07 | 0.54 ± 0.45 | -0.04 ± 0.06 | -0.04 ± 0.09 |
| | Iron 2x | 0.07 ± 0.07 | -0.06± 0.06 | -0.08 ± 0.08 | 0.47 ± 0.28 | -0.05 ± 0.17 | -0.04 ± 0.13 |
| | Iron 1x bLF 0.5x | -0.08 ± 0.06 | -0.08 ± 0.06 | -0.06 ± 0.04 | 0.23 ± 0.33 | 0.02 ± 0.05 | -0.18 ± 0.08 |
| | Iron 1x bLF 1x | 0.16 ± 0.20 | -0.03 ± 0.03 | -0.13 ± 0.10 | 0.63 ± 0.38 | **0.08 ± 0.05 *** | -0.18 ± 0.13 |
| | Iron 1x bLF 2x | 0.13 ± 0.09 | -0.04 ± 0.03 | -0.05 ± 0.09 | 0.64 ± 0.29 | 0.06 ± 0.05 | -0.16 ± 0.12 |
| | Iron 1x GOS 0.12x | 0.20 ± 0.19 | 0.00 ± 0.03 | 0.10 ± 0.15 | 0.50 ± 0.41 | 0.07 ± 0.12 | -0.04 ± 0.18 |
| | Iron 1x GOS 0.25x | 0.00 ± 0.11 | -0.11 ± 0.09 | 0.15 ± 0.19 | 0.29 ± 0.30 | -0.27 ± 0.15 | -0.09 ± 0.23 |
| | Iron 1x GOS 0.5x | 0.07 ± 0.11 | -0.12 ± 0.03 | -0.02 ± 0.34 | 0.29 ± 0.26 | -0.45 ± 0.38 | -0.21 ± 0.26 |
| | Iron 1x bLF 0.5x GOS 0.12x | 0.01 ± 0.11 | -0.12 ± 0.03 | 0.09 ± 0.14 | 0.27 ± 0.40 | 0.00 ± 0.12 | -0.10 ± 0.17 |
| | Iron 1x bLF 1x GOS 0.25x | 0.05 ± 0.24 | -0.05 ± 0.02 | 0.10 ± 0.20 | 0.20 ± 0.40 | -0.06 ± 0.15 | -0.19 ± 0.23 |
| | Iron 1x bLF 2x GOS 0.5x | 0.03 ± 0.25 | -0.09 ± 0.01 | 0.06 ± 0.33 | 0.03 ± 0.31 | -0.22 ± 0.32 | -0.33 ± 0.20 |

| | | *C. difficile* (bacteria/mL) | | | *C. perfringens* (bacteria/mL) | | | *EPEC* (bacteria/mL) | | |
|---|---|---|---|---|---|---|---|---|---|---|
| | | Donor 1 | Donor 2 | Donor 3 | Donor 1 | Donor 2 | Donor 3 | Donor 1 | Donor 2 | Donor 3 |
| $log_{10}$ | Inoculum | 8.25 ± 0.37 | 8.04 ± 0.22 | ND | 6.27 ± 0.53 | BDL | BDL | BDL | BDL | ND |
| | Control | 8.35 ± 0.49 | 8.44 ± 0.17 | 8.90 ± 0.19 | 8.68 ± 0.35 | BDL | BDL | BDL | BDL | 9.65 ± 0.13 |
| $\Delta log_{10}$ compared to control | Iron 0.5x | 0.30 ± 0.38 | -0.03 ± 0.29 | -0.07 ± 0.09 | 0.28 ± 0.22 | BDL | BDL | BDL | BDL | -0.03 ± 0.12 |
| | Iron 1x | 0.36 ± 0.36 | -0.19 ± 0.26 | -0.05 ± 0.11 | 0.30 ± 0.38 | BDL | BDL | BDL | BDL | -0.06 ± 0.12 |
| | Iron 2x | 0.21 ± 0.22 | -0.16 ± 0.45 | -0.11 ± 0.13 | 0.27 ± 0.33 | BDL | BDL | BDL | BDL | -0.08 ± 0.12 |
| | Iron 1x bLF 0.5x | 0.05 ± 0.54 | -0.27 ± 0.10 | -0.16 ± 0.10 | -0.06 ± 0.35 | BDL | BDL | BDL | BDL | -0.21 ± 0.10 |
| | Iron 1x bLF 1x | 0.29 ± 0.49 | -0.21 ± 0.15 | -0.21 ± 0.19 | 0.06 ± 0.35 | BDL | BDL | BDL | BDL | -0.20 ± 0.13 |
| | Iron 1x bLF 2x | 0.18 ± 0.34 | -0.14 ± 0.31 | -0.26 ± 0.12 | 0.26 ± 0.30 | BDL | BDL | BDL | BDL | -0.21 ± 0.13 |
| | Iron 1x GOS 0.12x | 0.21 ± 0.27 | -0.13 ± 0.17 | 0.01 ± 0.23 | 0.28 ± 0.51 | BDL | BDL | BDL | BDL | 0.10 ± 0.15 |
| | Iron 1x GOS 0.25x | 0.13 ± 0.51 | 0.25 ± 0.05 | -0.27 ± 0.42 | 0.56 ± 0.29 | BDL | BDL | BDL | BDL | -0.17 ± 0.29 |
| | Iron 1x GOS 0.5x | 0.26 ± 0.59 | 0.14 ± 0.40 | -0.61 ± 0.58 | 0.50 ± 0.56 | BDL | BDL | BDL | BDL | -0.31 ± 0.35 |
| | Iron 1x bLF 0.5x GOS 0.12x | -0.03 ± 0.17 | -0.27 ± 0.13 | -0.19 ± 0.33 | -0.03 ± 0.13 | BDL | BDL | BDL | BDL | -0.16 ± 0.24 |
| | Iron 1x bLF 1x GOS 0.25x | 0.01 ± 0.38 | 0.04 ± 0.32 | -0.30 ± 0.50 | -0.04 ± 0.13 | BDL | BDL | BDL | BDL | -0.21 ± 0.32 |
| | Iron 1x bLF 2x GOS 0.5x | -0.14 ± 0.51 | 0.08 ± 0.16 | -0.76 ± 0.58 | 0.16 ± 0.30 | BDL | BDL | BDL | BDL | -0.41 ± 0.30 |

Mean ± SD of $log_{10}$ bacteria/mL effluent is shown for inoculum (effluent) and control. The difference between treatments and control was calculated and is shown as mean ± SD of Δ metabolite concentration (ΔmM). n = 3 repeats per donor with inoculum derived from 3 consecutive fermentation days. Significant differences between treatments with GOS or bLF and iron 1x. * $p<0.05$. BDL: Below the detection limit.

**Supplementary Table 6**. Quantification of intermediate metabolites succinate and lactate and branched-SCFA isobutyrate and isovalerate in Kenyan infant PolyFermS microbiota treated with iron, GOS and bLF during 24 h batch fermentations in 24-well plates.

| | | Succinate (mM) | | | Lactate (mM) | | | Isobutyrate (mM) | | | Isovalerate (mM) | | |
|---|---|---|---|---|---|---|---|---|---|---|---|---|---|
| | | **Donor 1** | **Donor 2** | **Donor 3** | **Donor 1** | **Donor 2** | **Donor 3** | **Donor 1** | **Donor 2** | **Donor 3** | **Donor 1** | **Donor 2** | **Donor 3** |
| mM | Inoculum | BDL | 0.26 ± 0.24 | ND | 1.67 ± 0.42 | BDL | ND | BDL | BDL | ND | 0.87 ± 0.11 | 0.85 ± 0.90 | ND |
| | Control | 3.09 ± 0.42 | 2.69 ± 0.19 | 2.72 ± 0.37 | BDL | BDL | 2.99 ± 0.39 | BDL | BDL | 1.04 ± 0.11 | 1.10 ± 0.32 | 0.51 ± 0.20 | 1.06 ± 0.03 |
| ΔmM compared to control | Iron 0.5x | -0.10 ± 0.03 | -0.04 ± 0.11 | 0.32 ± 0.04 | BDL | BDL | 0.32 ± 0.34 | BDL | BDL | -0.03 ± 0.05 | 0.03 ± 0.02 | 0.16 ± 0.30 | 0.01 ± 0.02 |
| | Iron 1x | -0.17 ± 0.53 | -0.08 ± 0.10 | 0.21 ± 0.09 | BDL | BDL | 0.18 ± 0.48 | BDL | BDL | -0.04 ± 0.08 | 0.09 ± 0.20 | 0.21 ± 0.33 | 0.02 ± 0.01 |
| | Iron 2x | -0.27 ± 0.22 | -0.04 ± 0.11 | 0.17 ± 0.14 | BDL | BDL | 0.18 ± 0.34 | BDL | BDL | 0.02 ± 0.05 | 0.23 ± 0.03 | 0.30 ± 0.32 | 0.05 ± 0.03 |
| | Iron 1x bLF 0.5x | -0.33 ± 0.08 | 0.00 ± 0.04 | 0.53 ± 0.06 | BDL | BDL | 0.11 ± 0.20 | BDL | BDL | 0.01 ± 0.02 | 0.05 ± 0.23 | -0.02 ± 0.11 | 0.05 ± 0.03 |
| | Iron 1x bLF 1x | -0.42 ± 0.14 | -0.04 ± 0.06 | 0.74 ± 0.13 | BDL | BDL | 0.25 ± 0.26 | BDL | BDL | 0.01 ± 0.02 | 0.12 ± 0.33 | 0.20 ± 0.19 | 0.06 ± 0.04 |
| | Iron 1x bLF 2x | -0.13 ± 0.14 | 0.01 ± 0.03 | 1.19 ± 0.43 | BDL | BDL | 0.14 ± 0.38 | BDL | BDL | **0.10 ± 0.02 *** | 0.22 ± 0.19 | 0.41 ± 0.31 | 0.36 ± 0.10 |
| | Iron 1x GOS 0.12x | 0.55 ± 0.33 | 0.39 ± 0.15 | 1.13 ± 0.14 | BDL | BDL | 1.08 ± 0.23 | BDL | BDL | -0.01 ± 0.12 | 0.00 ± 0.15 | -0.15 ± 0.20 | -0.10 ± 0.03 |
| | Iron 1x GOS 0.25x | 0.82 ± 0.51 | 0.58 ± 0.18 | **2.25 ± 0.40 **** | BDL | BDL | **2.94 ± 0.42 **** | BDL | BDL | -0.08 ± 0.18 | -0.16 ± 0.03 | -0.35 ± 0.18 | -0.19 ± 0.11 |
| | Iron 1x GOS 0.5x | 0.78 ± 0.81 | 0.41 ± 0.29 | **2.97 ± 0.87 ***** | BDL | BDL | **9.39 ± 1.54 ****** | BDL | BDL | -0.37 ± 0.33 | -0.21 ± 0.41 | -0.42 ± 0.23 | -0.47 ± 0.13 |
| | Iron 1x bLF 0.5x GOS 0.12x | -0.18 ± 1.13 | -0.39 ± 0.38 | **1.25 ± 0.33 *** | BDL | BDL | 0.79 ± 0.52 | BDL | BDL | 0.03 ± 0.11 | -0.16 ± 0.17 | -0.21 ± 0.02 | -0.09 ± 0.05 |
| | Iron 1x bLF 1x GOS 0.25x | -0.44 ± 1.03 | -0.24 ± 0.27 | **2.38 ± 0.47 **** | BDL | BDL | **2.75 ± 0.99 **** | BDL | BDL | -0.13 ± 0.11 | **-0.34 ± 0.13 *** | -0.36 ± 0.19 | -0.16 ± 0.09 |
| | Iron 1x bLF 2x GOS 0.5x | -0.05 ± 1.14 | -0.08 ± 0.39 | **3.00 ± 0.74 ***** | BDL | BDL | **6.98 ± 0.86 ****** | BDL | BDL | **-0.41 ± 0.35 *** | **-0.57 ± 0.18 **** | **-0.45 ± 0.11 *** | -0.24 ± 0.21 |

Mean ± SD of absolute metabolite concentration (mM) is shown for inoculum (IR effluent) and control. The difference between treatments and control was calculated and is shown as mean ± SD of Δ metabolite concentration (ΔmM). n = 3 repeats per donor with inoculum derived from 3 consecutive fermentation days. Significant differences between iron 1x and the GOS and bLF treatments indicated by * $p<0.05$, ** $p<0.01$, *** $p<0.001$, **** $p<0.0001$. ND: not determined, BDL: below the detection limit.

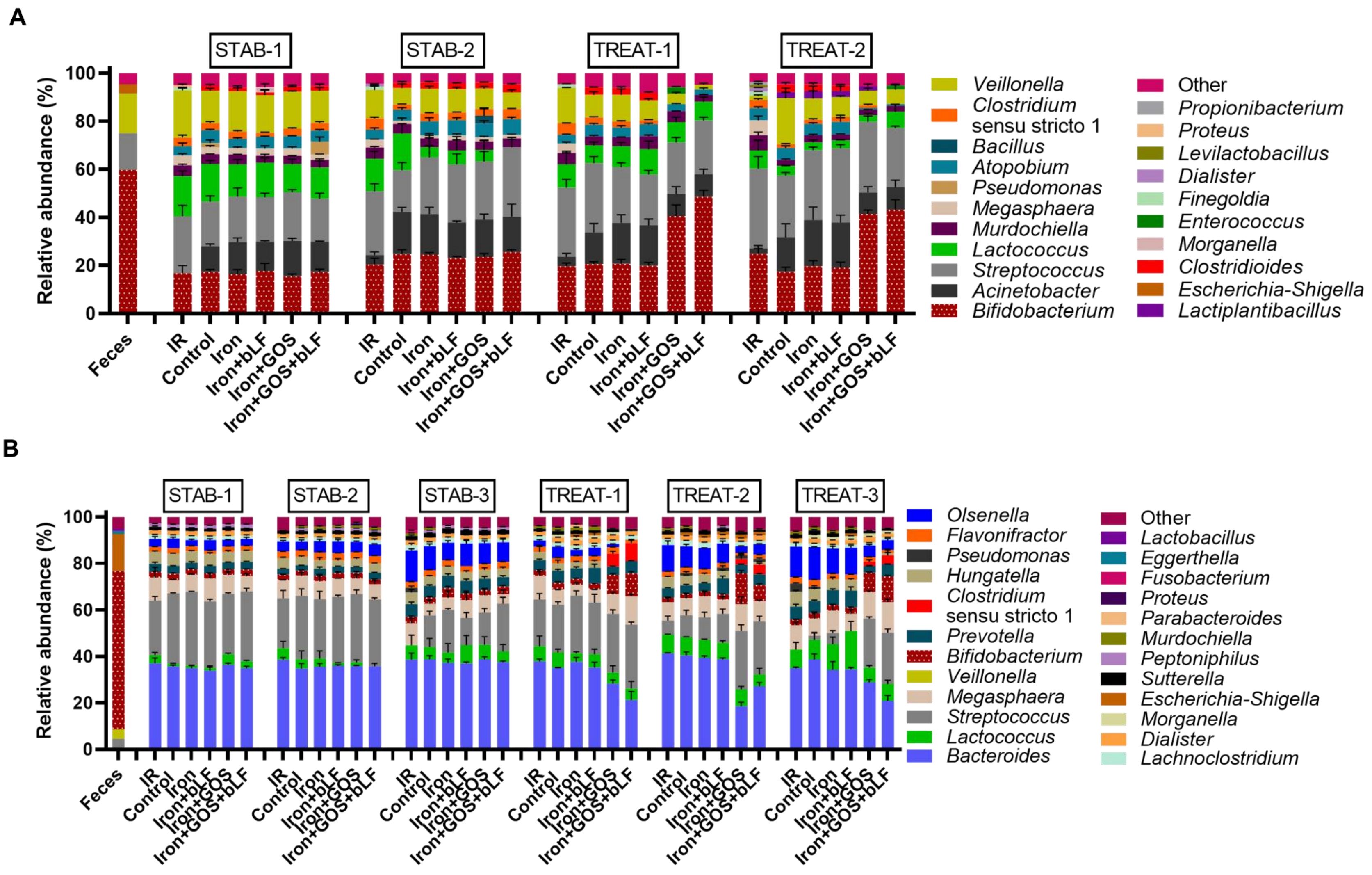


**Supplementary Figure 3.** Microbiota composition of feces, IR and TRs at the end of stabilization and treatment period measured. Relative abundance on genus level («other»: <1%) of donor 4 (**A**) and donor 5 (**B**). Mean ± SD of the last three days of stabilization (STAB) and the last three days of treatment (TREAT) is shown for each experimental period.

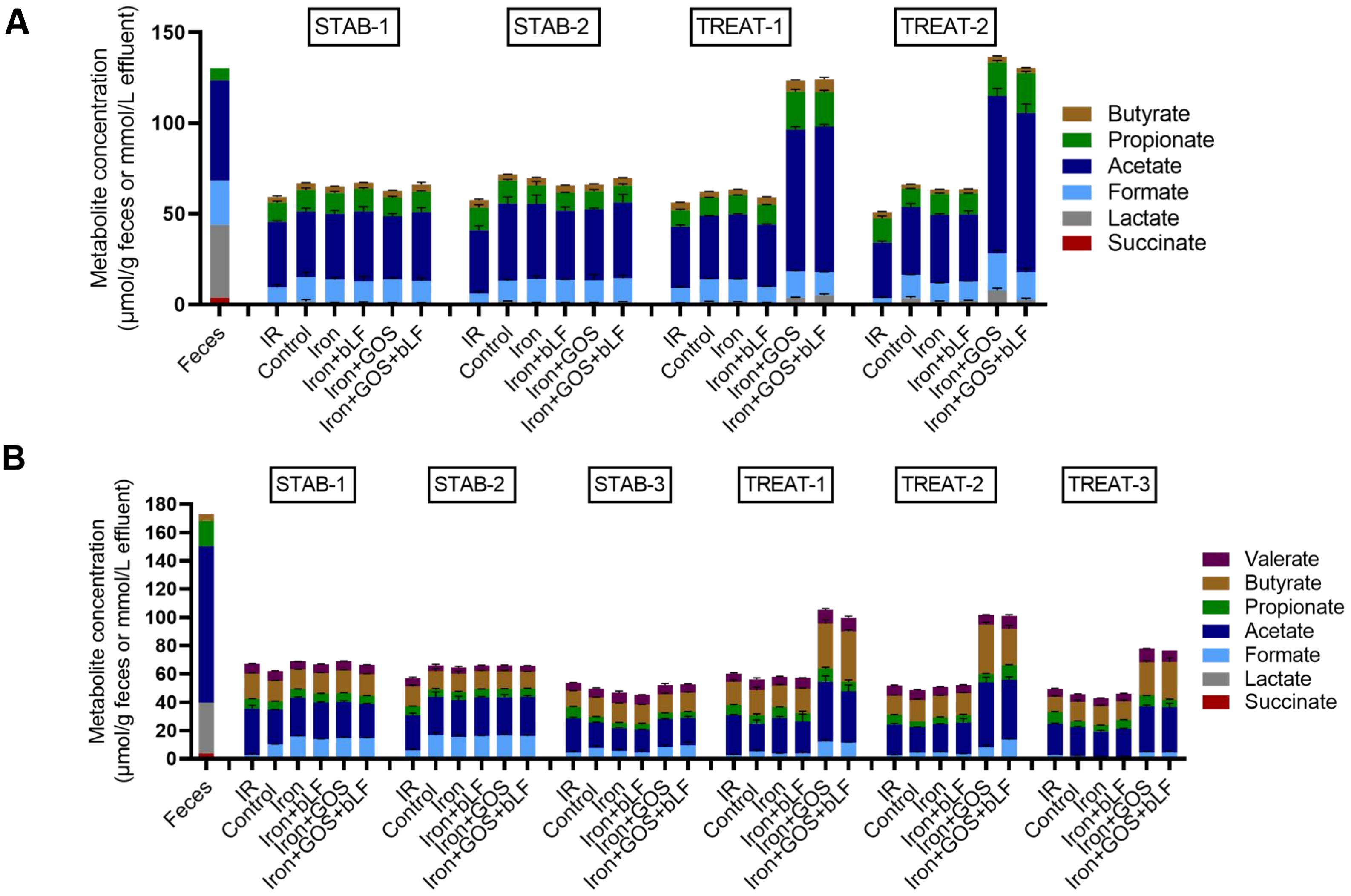


**Supplementary Figure 4.** Metabolite profile of feces, IR and TRs at the end of stabilization and treatment period. Concentrations of SCFA, BCFA and intermediate fermentation metabolites of donor 4 (**A**) and donor 5 (**B**). Mean ± SD of the last three days of stabilization (STAB) and the last three days of treatment (TREAT) is shown for each experimental period.

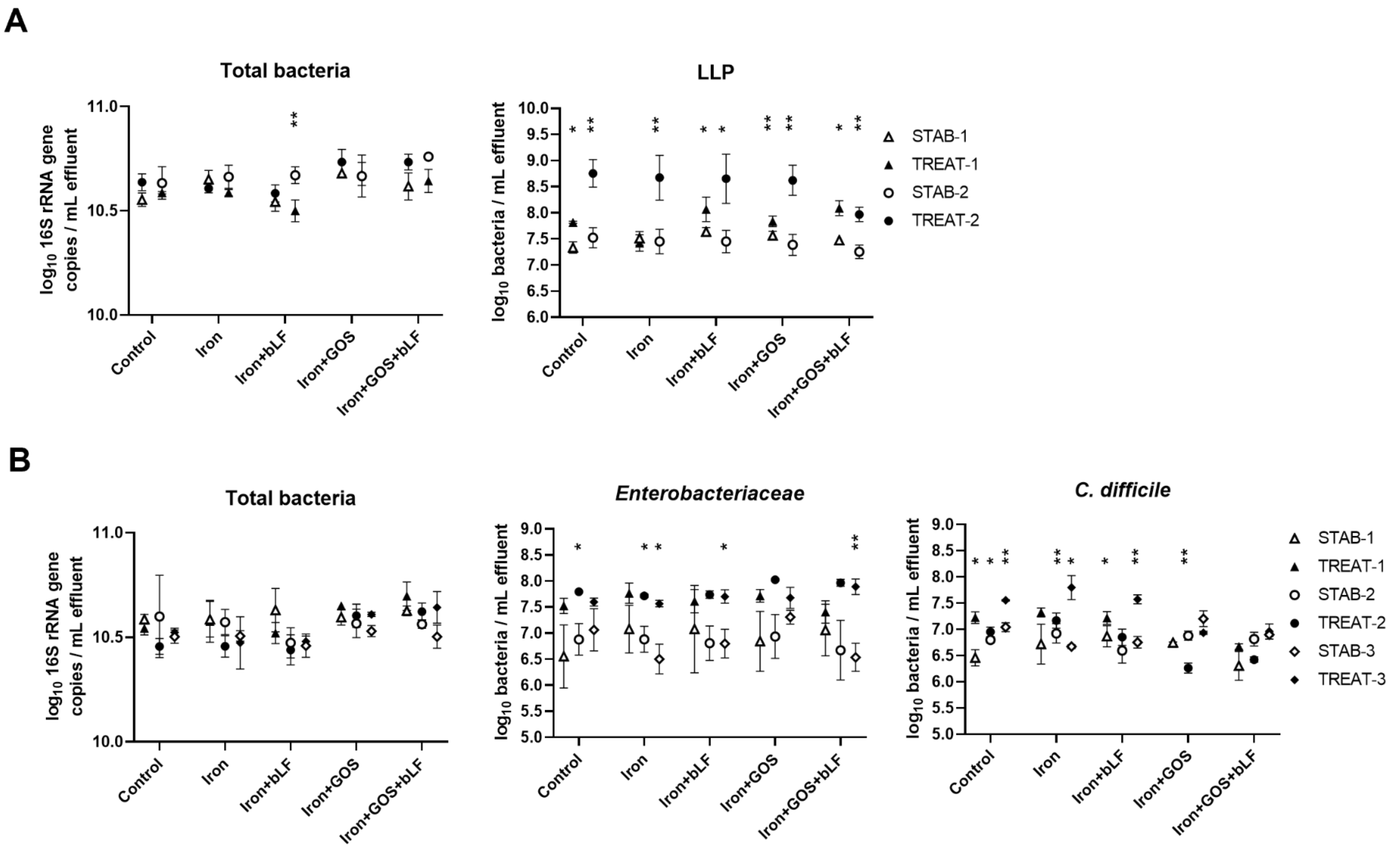


**Supplementary Figure 5.** Quantification of beneficial and potential pathogenic taxa before and after treatment with iron, GOS and bLF. Mean ± SD of 16S rRNA gene copies or bacteria/mL effluent is shown for the last three days of stabilization (STAB) and the last three days of treatment (TREAT) of two experimental periods of donor 4 (**A**) and three experimental periods of donor 5 (**B**). Significant differences between STAB and TREAT of corresponding periods are indicated by * $p<0.05$, ** $p<0.01$.

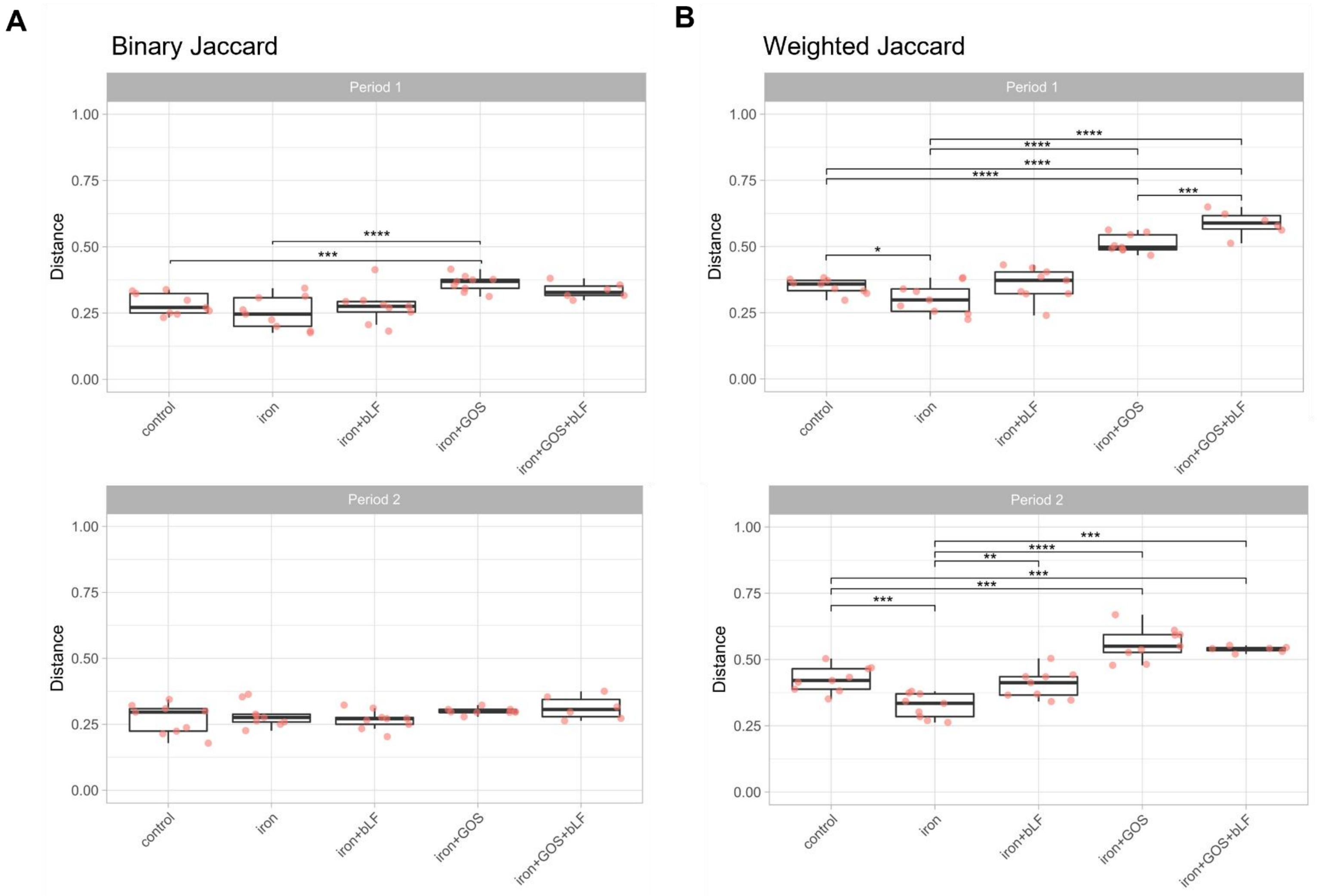


**Supplementary figure 6.** Distance boxplots for shifts in continuous PolyFermS Kenyan infant microbiota composition of donor 4 after treatment with iron, GOS and bLF based on 16S rRNA gene amplicon sequencing data. Binary **(A)** and weighted **(B)** Jaccard distance between the microbiota at the end of stabilization and the microbiota at the end of treatment shown for each experimental period. Significant differences in compositional shifts between control or iron and all other treatments are indicated, as well as between iron+GOS and iron+GOS+bLF. * $p<0.05$, ** $p<0.01$, *** $p<0.001$, **** $p<0.0001$

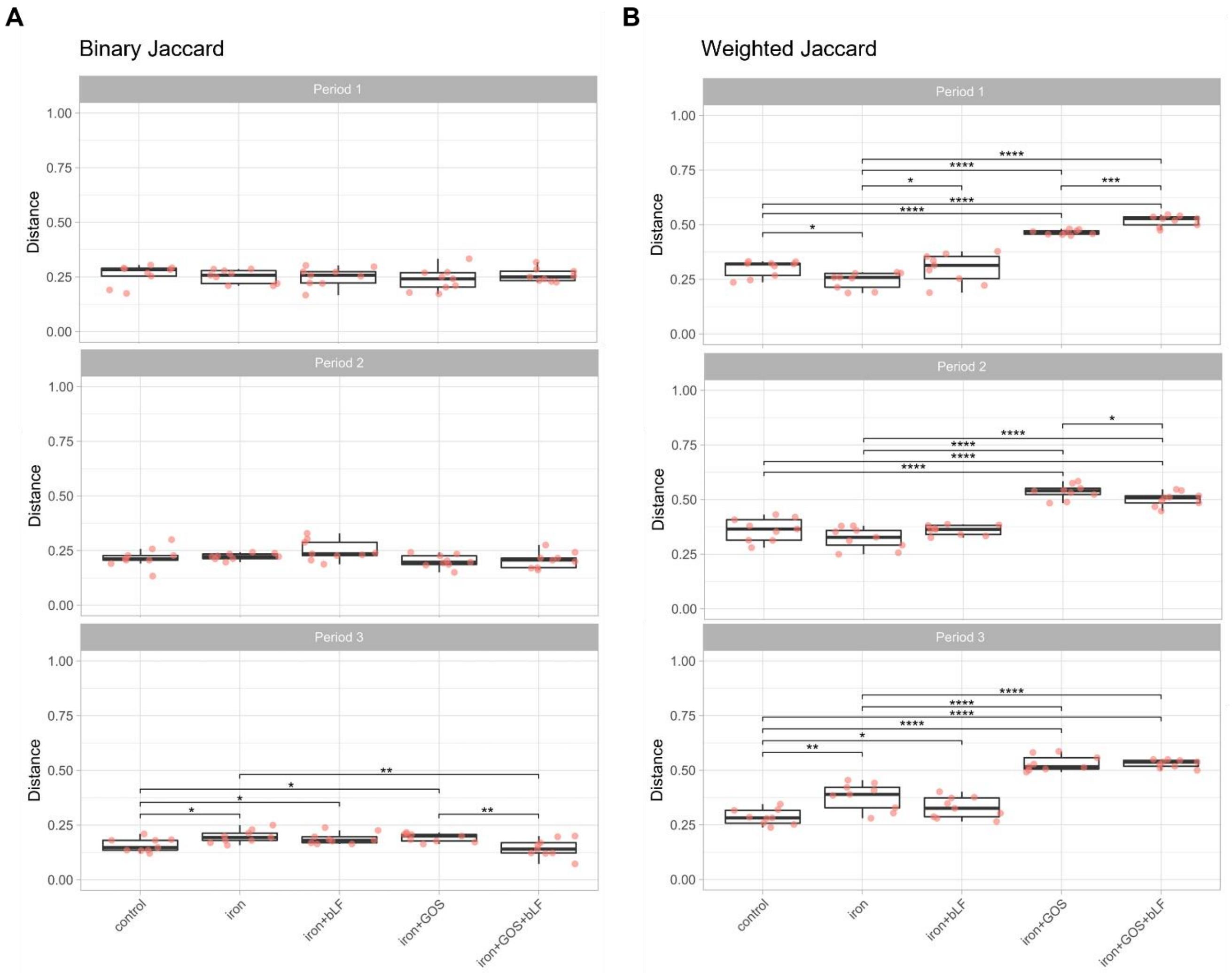


**Supplementary Figure 7.** Distance boxplots for shifts in continuous PolyFermS Kenyan infant microbiota composition of donor 5 after treatment with iron, GOS and bLF based on 16S rRNA gene amplicon sequencing data. Binary **(A)** and weighted **(B)** Jaccard distance between the microbiota at the end of stabilization and the microbiota at the end of treatment shown for each experimental period. Significant differences in compositional shifts between control or iron and all other treatments are indicated, as well as between iron+GOS and iron+GOS+bLF. * $p<0.05$, ** $p<0.01$, *** $p<0.001$, **** $p<0.0001$

**A**

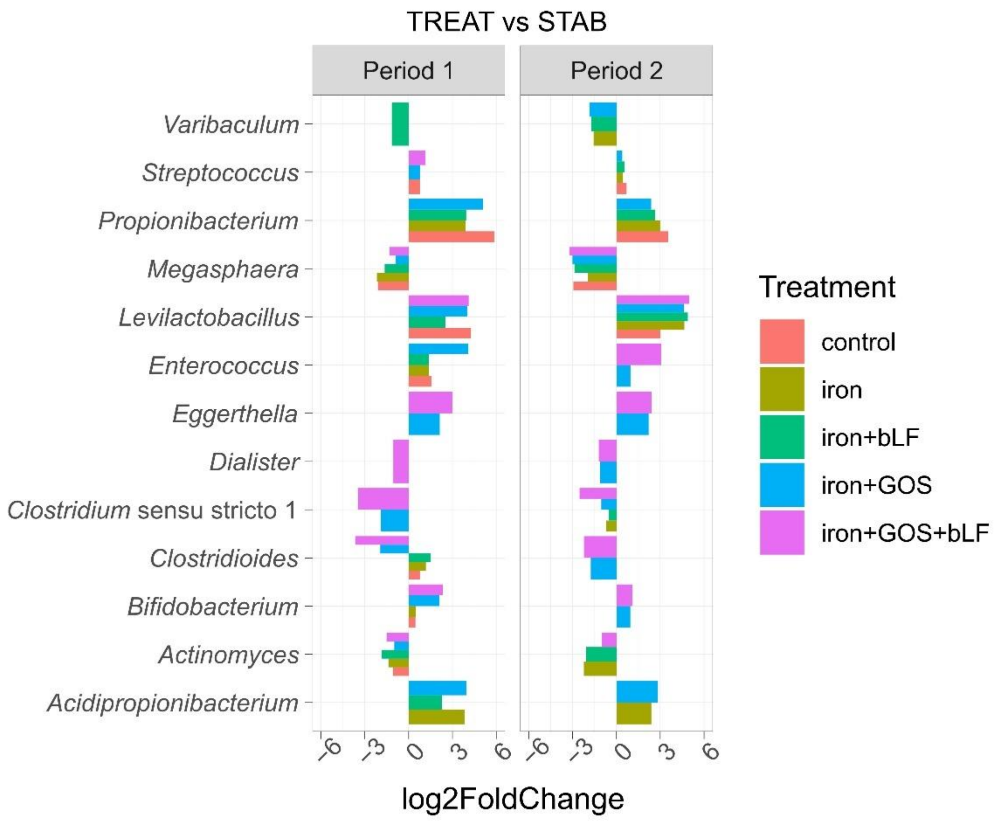


**B**

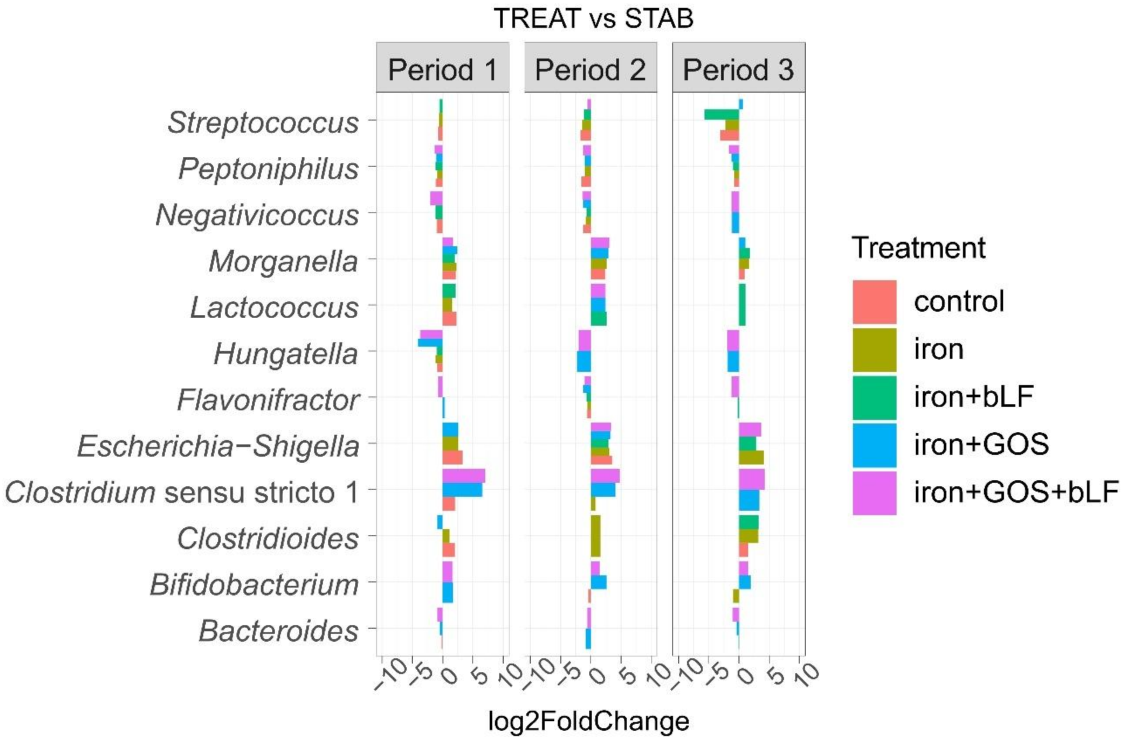


**Supplementary Figure 8.** Differential abundance analysis at genus level of continuous PolyFermS Kenyan infant microbiota after treatment with iron, GOS and bLF with DESeq2. Barplots show $log_2$-fold changes of genera significantly ($p<0.05$) different in relative abundance between the last three days of stabilization and the last three days of treatment for all experimental periods in donor 4 **(A)** and donor 5 microbiota **(B)**.

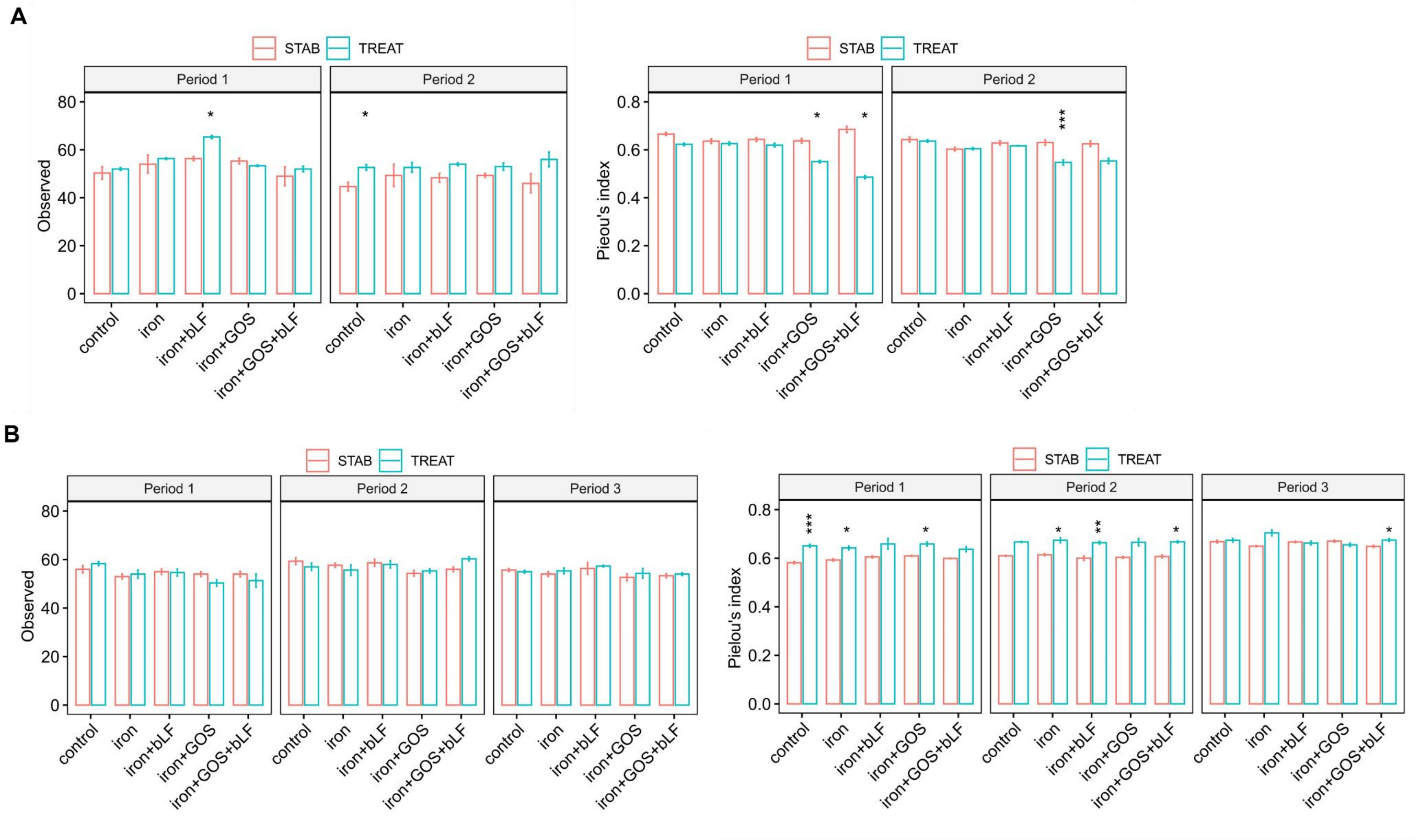


**Supplementary Figure 9.** Alpha diversity of continuous PolyFermS Kenyan infant microbiota after treatment with iron, GOS and bLF. The number of observed ASVs and Pielou's evenness index are shown for donor 4 (**A**) and donor 5 microbiota (**B**). Differences were assessed between the last three days of stabilization (STAB) and the last three days of treatment (TREAT) of each experimental period. *p<0.05, ** p<0.01, *** p<0.001

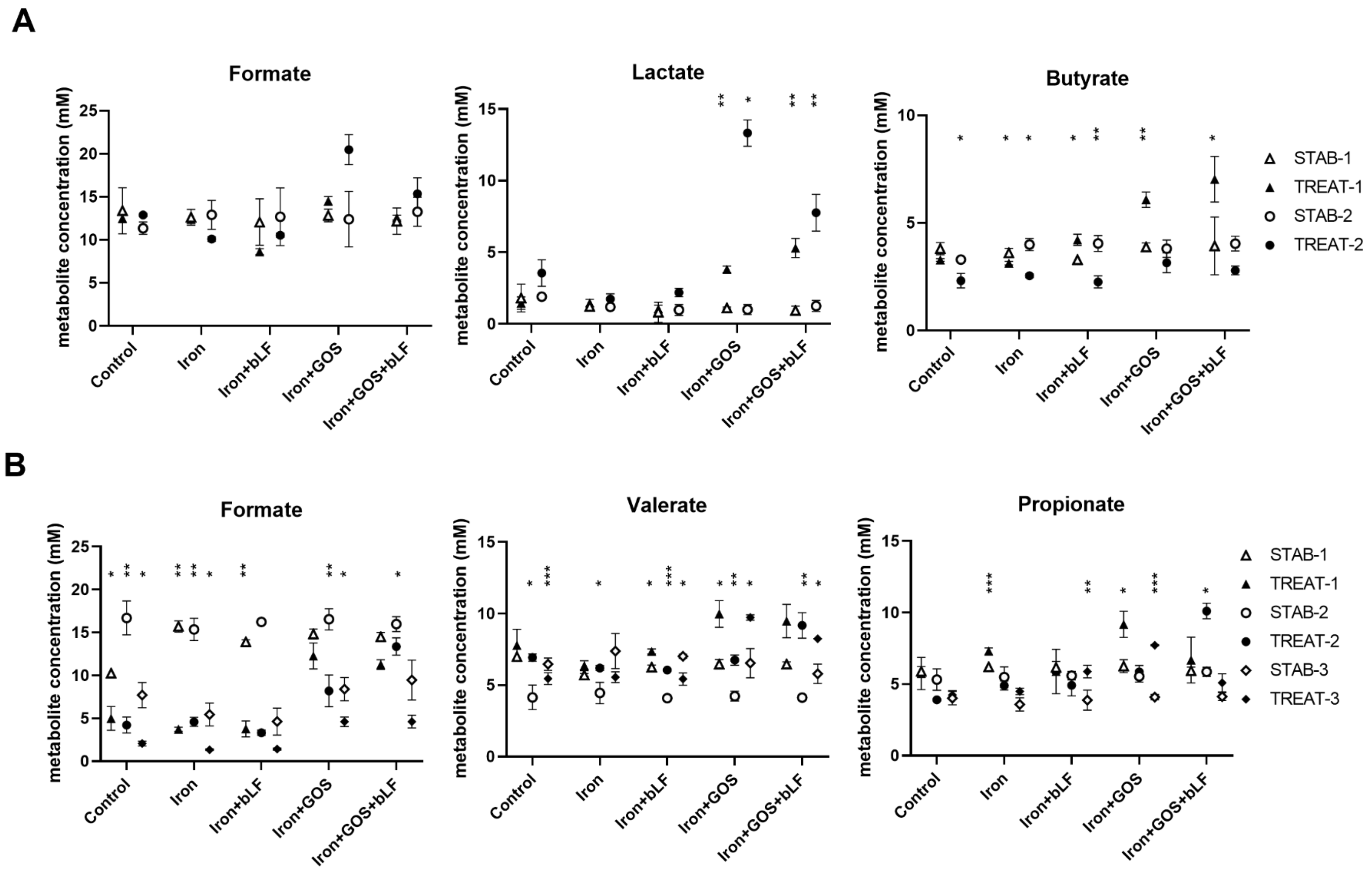


**Supplementary figure 10.** Concentration of total and intermediate metabolites, and SCFA before and after treatment of continuous PolyFermS Kenyan infant microbiota with iron, GOS and bLF. Mean ± SD of metabolite concentration is shown for the last three days of stabilization (STAB) and the last three days of treatment (TREAT) of two experimental periods of donor 4 **(A)** and three experimental periods of donor 5 **(B)**. Significant differences between STAB and TREAT in the corresponding periods are indicated. * $p<0.05$, ** $p<0.01$, *** $p<0.001$

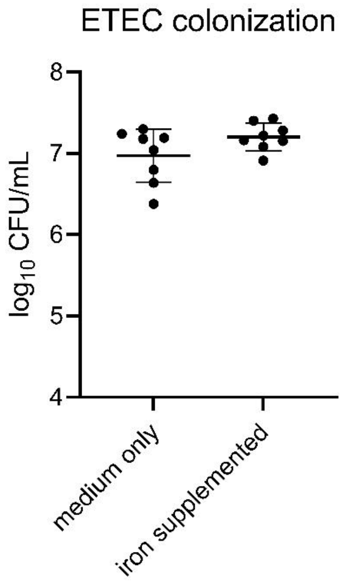


**Supplementary Figure 11.** Colony forming units (CFU) of adhered and invaded ETEC in a Caco-2/HT29-MTX/THP-1 Blue cell co-culture model after 3 h of infection in the presence of mammalian cell cultivation medium only or supplemented with 5 mg/L iron.

**Supplementary Table 7.** Relative abundance of genera with significant correlations to assessed cellular parameters.

| | Donor 4 | | Donor 5 | | | |
|---|---|---|---|---|---|---|
| | *Pseudomonas* | *Finegoldia* | *Bacteroides* | *Hungatella* | *Flavonifractor* | *Lachno clostridium* |
| Control | 0.19% | 0.95% | 40.30% | 4.42% | 2.50% | 2.72% |
| Iron | 0.09% | 0.87% | 38.58% | 4.37% | 2.10% | 2.49% |
| Iron+bLF | 0.12% | 0.76% | 38.31% | 3.31% | 1.96% | 1.92% |
| Iron+GOS | 0.00% | 0.75% | 18.82% | 0.56% | 1.13% | 1.32% |
| Iron+GOS+bLF | 0.02% | 0.47% | 27.36% | 0.95% | 1.56% | 1.44% |

## References


1. C C, G LB, I F, C L. Immobilization of infant fecal microbiota and utilization in an in vitro colonic fermentation model. *Microb Ecol* 2004;48:128–38. [PMID: 15085302 DOI: 10.1007/S00248-003-2022-7]

2. Horniblow RD, Mistry P, Quraishi MN, Beggs AD, Van De Wiele T, Iqbal TH, et al. The safety and tolerability of a potential alginate-based iron chelator; results of a healthy participant study. *Nutrients* 2019;11:1–14. [DOI: 10.3390/nu11030674]

3. Phillips E, Horniblow RD, Poole V, Bedford M, Ward DG, Kirkham AJ, et al. Potential role for hepcidin in obesity-driven colorectal tumourigenesis. *Oncol Rep* 2018;39:392–400. [PMID: 29115635 DOI: 10.3892/OR.2017.6062/HTML]

4. Fierer N, Jackson JA, Vilgalys R, Jackson RB. Assessment of soil microbial community structure by use of taxon-specific quantitative PCR assays. *Appl Environ Microbiol* 2005;71:4117–20. [PMID: 16000830 DOI: 10.1128/AEM.71.7.4117-4120.2005]

5. Bartosch S, Fite A, Macfarlane GT, McMurdo MET. Characterization of bacterial communities in feces from healthy elderly volunteers and hospitalized elderly patients by using real-time PCR and effects of antibiotic treatment on the fecal microbiota. *Appl Environ Microbiol* 2004;70:3575–81. [PMID: 15184159 DOI: 10.1128/AEM.70.6.3575-3581.2004]

6. Furet J-P, Firmesse O, Gourmelon M, Bridonneau C, Tap J, Mondot S, et al. Comparative assessment of human and farm animal faecal microbiota using real-time quantitative PCR. *FEMS Microbiol Ecol* 2009;68:351–62. [PMID: 19302550 DOI: 10.1111/j.1574-6941.2009.00671.x]

7. Rinttilä T, Kassinen A, Malinen E, Krogius L, Palva A. Development of an extensive set of 16S rDNA-targeted primers for quantification of pathogenic and indigenous bacteria in faecal samples by real-time PCR. *J Appl Microbiol* 2004;97:1166–77. [PMID: 15546407 DOI: 10.1111/j.1365-2672.2004.02409.x]

8. Fukushima H, Tsunomori Y, Seki R. Duplex real-time SYBR green PCR assays for detection of 17 species of food- or waterborne pathogens in stools. *J Clin Microbiol* 2003;41:5134–46. [PMID: 14605150 DOI: 10.1128/JCM.41.11.5134-5146.2003]
